# Polygenic Score Approach to Predicting Risk of Metabolic Syndrome

**DOI:** 10.3390/genes16010022

**Published:** 2024-12-26

**Authors:** Yanina Timasheva, Olga Kochetova, Zhanna Balkhiyarova, Gulnaz Korytina, Inga Prokopenko, Arie Nouwen

**Affiliations:** 1 Institute of Biochemistry and Genetics, Ufa Federal Research Centre of Russian Academy of Sciences, 450054 Ufa, Russia; yartimasheva@bashgmu.ru (Y.T.); ovkochetova@bashgmu.ru (O.K.); gfkorytina@bashgmu.ru (G.K.); 2 Department of Medical Genetics and Fundamental Medicine, Faculty of General Medicine, Bashkir State Medical University, 450008 Ufa, Russia; 3 Department of Biology, Faculty of Stomatology, Bashkir State Medical University, 450008 Ufa, Russia; 4 Department of Endocrinology, Faculty of General Medicine, Bashkir State Medical University, 450008 Ufa, Russia; z.balkhiiarova@surrey.ac.uk; 5 Section of Statistical Multi-Omics, Department of Clinical & Experimental Medicine, School of Biosciences & Medicine, University of Surrey, Guildford GU2 7XH, UK; 6 Department of Psychology, Middlesex University, London NW4 4BT, UK

**Keywords:** metabolic syndrome, polygenic score, predictive medicine

## Abstract

Background/Objectives: Metabolic syndrome (MetS) is a complex condition linking obesity, diabetes, and hypertension, representing a major challenge in clinical care. Its rising global prevalence, driven by urbanization, sedentary lifestyles, and dietary changes, underscores the need for effective management. This study aims to explore the genetic mechanisms behind MetS, including adiposity, inflammation, neurotransmitters, and β-cell function, to develop a prognostic tool for MetS risk. Methods: We genotyped 40 genetic variants across these pathways in 279 MetS patients and 397 healthy individuals. Using logistic regression, we evaluated the prognostic capability of a polygenic score model for MetS risk, both independently and with other factors like sex and age. Results: Logistic regression analysis identified 18 genetic variants significantly associated with MetS. The optimal predictive model used polygenic scores calculated with weights assigned to the 18 loci (AUC 82.5%, 95% CI 79.4–85.6%), with age and sex providing a minimal, non-significant improvement (AUC 83.3%, 95% CI 80.2–86.3%). The addition of the polygenic score significantly improved net reclassification (NRI = 1.03%, *p* = 3.42 × 10^−50^). Including all 40 variants did not enhance prediction (NRI = −0.11, *p* = 0.507). Conclusions: Polygenic scores could aid in predicting MetS risk and health outcomes, emphasizing the need for diagnostic tools tailored to specific populations. Additional research is warranted to corroborate these conclusions and explore the molecular mechanisms of MetS.

## 1. Introduction

Metabolic syndrome (MetS) encompasses a spectrum of interdependent pathophysiological conditions, which share common underlying mechanisms and mutually reinforce one another. These include visceral obesity, dyslipidemia, elevated blood pressure (BP), and altered glucose tolerance [1]. The global prevalence of MetS is rapidly increasing, driven not only by genetic predisposition but also by significant lifestyle changes associated with urbanization. The transition to urban environments often brings dietary shifts toward high-calorie, processed foods, reduced physical activity due to sedentary work and leisure habits, and greater life expectancies, all of which contribute to the rising burden of MetS [2]. The importance of MetS lies in its high prevalence and its association with elevated risks for type 2 diabetes (T2D) and its complications and coronary artery disease (CAD), along with its adverse effects on both longevity and health-related quality of life. Given the complexity of MetS, research often focuses on genes linked to adiposity, lipid metabolism, diabetes, and glycemic traits [3,4,5]. Previously, we identified several genes encoding neurotransmitters and inflammatory mediators associated with MetS and related conditions [6,7,8,9].

Subclinical inflammation is a critical hallmark of MetS, frequently indicated by increased concentrations of biomarkers including high-sensitivity C-reactive protein (hsCRP) [10], tumor necrosis factor receptor 1 (TNFR1), soluble tumor necrosis factor receptor 2 (sTNFR2), and leptin (LEP) [11]. Our recent research has highlighted associations between *TNFSF1B* rs1061624 and MetS [12]. Additionally, *TNF* rs1800629 has been linked to elevated TNF-α levels and albuminuria, an indicator of diabetic nephropathy [10]. Similarly, *CRP* rs2794521 has shown associations with hsCRP and a range of anthropometric and metabolic traits, including height, body mass index (BMI), waist-to-hip ratio (WHR), postprandial glucose, fasting insulin, and homeostatic model assessment of insulin resistance (HOMA-IR) [13]. Associations with MetS have also been observed for *TMEM18* rs2860323 [14].

Recent genome-wide association studies (GWASs) have pinpointed loci linked to various metabolic phenotypes, further supporting the potential of polygenic risk scoring in predicting susceptibility to MetS [3]. Polygenic scores, which assess an individual’s genetic predisposition, have effectively demonstrated genetic susceptibility to MetS in children and adolescents, and even examined the interaction between adherence to a Mediterranean diet, adiposity, and MetS risk [15,16,17].

This study aims to leverage data on genetic associations within 40 key genes related to adiposity, inflammation, β-cell function, and neurotransmitter activity to construct a polygenic risk score that can predict susceptibility to MetS and its endophenotypes.

## 2. Materials and Methods

### 2.1. Study Group

This study was carried out following the principles outlined in the Declaration of Helsinki and received approval from the Local Ethical Committee of the Institute of Biochemistry and Genetics UFRC RAS (Protocol No 8, 14 March 2012). All participants provided their written informed consent.

This study involved 279 individuals with metabolic syndrome (MetS) and 397 healthy controls. The recruitment process and enrollment criteria are detailed elsewhere [8,12]. Briefly, the MetS criteria included a waist circumference exceeding 102 cm for men and 88 cm for women, blood pressure (BP) above 130/85 mmHg, fasting triglycerides (TGs) exceeding 1.7 mmol/L, fasting high-density lipoprotein (HDL) cholesterol below 1.03 mmol/L for men or 1.3 mmol/L for women, and fasting glucose (FG) above 5.6 mmol/L [7,18,19]. The control group participants exhibited no clinical or laboratory signs of metabolic conditions, no family history of diabetes, and were unrelated to the other study participants.

### 2.2. Clinical Measurements

The anthropometric measurements followed the WHO guidelines [20]. Body weight was recorded to the nearest kilogram with participants in light clothing, and height was measured to the nearest centimeter using a stadiometer. Waist circumference was assessed at the midpoint between the lower rib and iliac crest, and hip circumference was measured at the widest part of the hips. Body mass index (BMI) was calculated as weight in kilograms divided by height in squared meters (kg/m^2^), and the waist-to-hip ratio (WHR) was calculated as waist divided by hip circumference.

BP was taken three times on both arms after a five-minute rest, using a standard sphygmomanometer. Systolic (SBP) and diastolic blood pressure (DBP) were determined from Korotkoff sounds (phases I and V) [21]. Mean arterial pressure was calculated as two-thirds DBP plus one-third SBP, and pulse pressure was calculated as SBP minus DBP.

Fasting and postprandial blood samples were collected. Plasma glucose was measured via the glucose oxidase method, and insulin was measured by an electrochemiluminescence immunoassay. Insulin resistance was estimated using the homeostasis model assessment (HOMA-IR) [22]. HbA1c was assessed by high-performance liquid chromatography (ADAMS A1c HA-8182, Arkray, Inc., Kyoto, Japan), while lipid profiles (total cholesterol (TC), TGs, HDL, and low-density lipoprotein (LDL)), C-reactive protein (CRP), and tumor necrosis factor alpha (TNF-α) were measured using the standard photometric (Olympus, Hamburg, Germany), chemiluminescent (IMMULITE 2000, Siemens Medical Solutions Diagnostics, Deerfield, IL, USA), and ELISA (Vector-Best, Novosibirsk, Russia) methods. Additional biomarkers (albumin, alanine aminotransferase (ALT), aspartate aminotransferase (AST), gamma-glutamyl transferase (GGT), creatinine, uric acid) were analyzed on a Cobas Integra 400 plus system (Roche Diagnostics, Basel, Switzerland).

### 2.3. Genotyping

Peripheral blood samples were collected from all participants and processed for DNA extraction. DNA isolation procedures and genotyping were carried out using the established protocols referenced in previous studies [9,13,23,24,25,26,27,28,29]. The genetic variants included in this study were chosen based on the findings from phenome-wide association studies (PheWASs), which identified associations with metabolic traits, such as impaired glucose tolerance, atherosclerosis, adiposity, as well as related diseases, including inflammatory disorders. Genotyping was performed using real-time PCR (CFX96, Bio-Rad Laboratories, Hercules, CA, USA) with TaqMan assays (Thermo Fisher Scientific Inc., Waltham, MA, USA). To ensure data reliability, 5% of the genotyped samples were chosen at random for re-genotyping, with the results fully consistent with the original data.

### 2.4. Statistical Analysis

Associations between genetic variants and MetS traits, as well as clinical parameters, were evaluated using logistic or linear regression analyses under the additive genetic model with age and sex as covariates, implemented in PLINK 1.9 [30]. The additive model posits that the effect of carrying two risk alleles is double that of carrying one. To account for multiple testing, the false discovery rate (FDR) was managed using the Benjamini–Hochberg method [31], with statistical significance defined as a P_FDR_ value less than 0.05.

Polygenic scores (both weighted and unweighted) were constructed for the genetic variants that showed significant associations with MetS in the study cohort based on the logistic regression results. Weighted scores used odds ratios (ORs), adjusted for age and sex, to assign weight to risk alleles. If the initial ORs were less than 1.0, the analysis was re-conducted with the alternative allele as the reference allele. Linkage equilibrium of variants on the same chromosome was assessed, and single nucleotide variants (SNVs) in linkage disequilibrium were not included in the calculation of polygenic scores.

To investigate the biological mechanisms underlying metabolic syndrome (MetS) endophenotypes and identify gene clusters associated with MetS and its traits, we utilized publicly available data from the UK Biobank, accessed via the Edinburgh Gene Atlas and PheWAS (phenome-wide association study) databases [32]. These datasets provided association results for genetic variants and phenotypic traits. We used the pheatmap R package to visualize clusters of associations among the studied genetic loci [33]. In the generated heatmap, columns represent gene clusters, rows correspond to distinct metabolic endophenotypes, and cell intensities reflect Z-scores aligned to the effect allele. This approach captures both the strength and direction of the associations between genetic variants within each cluster and the metabolic endophenotypes.

The prognostic value of the derived polygenic scores for MetS was evaluated through Receiver Operator Characteristic (ROC) analysis. Model performance was quantified using the area under the ROC curve (AUC). Predictive models for MetS were developed using the Epi: Statistical Analysis in Epidemiology [34] and *pROC* [35] R packages.

To assess the enhancement in risk prediction provided by incorporating additional parameters, the net reclassification index (NRI) was calculated [36]. Continuous NRI values were obtained using the nribins function from the nricens: NRI for Risk Prediction Models with Time to Event and Binary Response Data R package [37]. Bootstrapping was employed to calculate 95% confidence intervals for NRI.

## 3. Results

### 3.1. Association Analysis

The association analysis revealed 18 SNVs significantly linked to metabolic syndrome (MetS) (Table 1; full results available in Supplementary Table S1). Notably, the strongest associations were observed for *CDKAL1* rs9295474*C (OR = 2.63 P_FDR_ = 1.79 × 10^−9^), *NPY2R* rs1047214*C (OR = 2.12, P_FDR_ = 1.50 × 10^−7^), *ADRA2A* rs1800544*G (OR = 2.18, P_FDR_ = 3.98 × 10^−6^), and *CHRM1* rs2067477*A (OR = 3.08 P_FDR_ = 3.0110^−5^).

Further analysis of the phenotypic traits in MetS patients highlighted significant associations with *ADCY3* rs17799872 across various characteristics. These include anthropometric traits like height (β = −3.86, P_FDR_ = 8.65 × 10^−6^), BMI (β = 2.16, P_FDR_ = 4.38 × 10^−13^), waist circumference (β = 6.19, P_FDR_ = 1.52 × 10^−6^), hip circumference (β = 3.99, P_FDR_ = 0.002), and waist–hip ratio (β = 0.02, P_FDR_ = 2.67 × 10^−4^). *ADCY3* rs17799872 was also strongly associated with glycemic traits, including fasting glucose (β = 0.59, P_FDR_ = 0.035), fasting insulin (β = 4.13, P_FDR_ = 2.14 × 10^−4^), postprandial insulin (β = 5.75, P_FDR_ = 0.001), and HOMA-IR (β = 1.48, P_FDR_ = 2.69 × 10^−4^). Additionally, *ADCY3* rs17799872 was associated with inflammatory markers such as erythrocyte sedimentation rate (β = 4.11, P_FDR_ = 0.049), CRP (β = 1.06, P_FDR_ = 1.10 × 10^−4^), TNF-α (β = 7.50, P_FDR_ = 0.001), fibrinogen (β = 0.76, P_FDR_ = 0.002), and albumin (β = −2.56, P_FDR_ = 0.002). Other loci remaining significant after multiple testing corrections include *ADRA2A* rs1800544, which was linked to glycemic traits like HOMA-IR (β = 0.79, P_FDR_ = 0.049) and postprandial insulin (β = 3.55, PFDR = 0.008), as well as *HTR2C* rs6318, associated with anthropometric traits including BMI (β = 1.29, P_FDR_ = 4.00 × 10^−4^), height (β = −2.82, P_FDR_ = 0.008), and waist circumference (β = 3.75, P_FDR_ = 0.048) (Supplementary Table S2).

**Figure 1:**
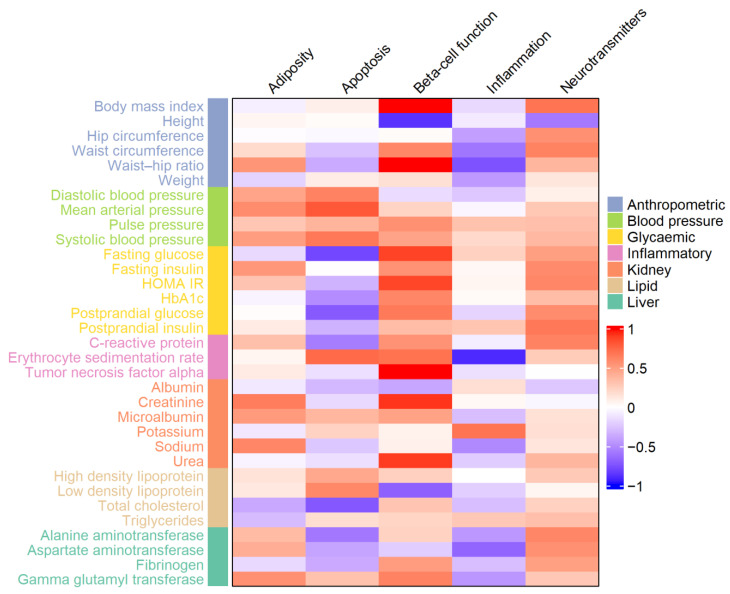
Heatmap illustrating the associations between 33 metabolic endophenotypes and five gene clusters linked to metabolic syndrome. Columns represent the gene clusters, and rows correspond to distinct metabolic endophenotypes. Each cell’s intensity reflects the Z-score, calculated based on linear regression analyses with adjustments for covariates, aligned to the effect allele. The Z-score indicates both the magnitude and direction of the association between the genetic variants in each cluster and the corresponding endophenotype.

To deepen our insight into the biological mechanisms driving metabolic syndrome (MetS) endophenotypes and to identify gene clusters associated with MetS and its traits, we analyzed publicly available data from the UK Biobank, accessed through the Edinburgh Gene Atlas and PheWAS (phenome-wide association study) databases [32]. These resources provided association results between the studied genetic variants and phenotypic traits, with the top associations highlighted in Supplementary Figure S1. Using these data, we visualized the clusters of associations for the studied genetic loci in a heatmap (Figure 1). In this representation, columns correspond to gene clusters, and rows represent distinct metabolic endophenotypes. The intensity of each cell reflects the Z-score, aligned to the effect allele, indicating both the strength and direction of the association between genetic variants within each cluster and the endophenotypes.

### 3.2. Polygenic Score Analysis

**Figure 2:**
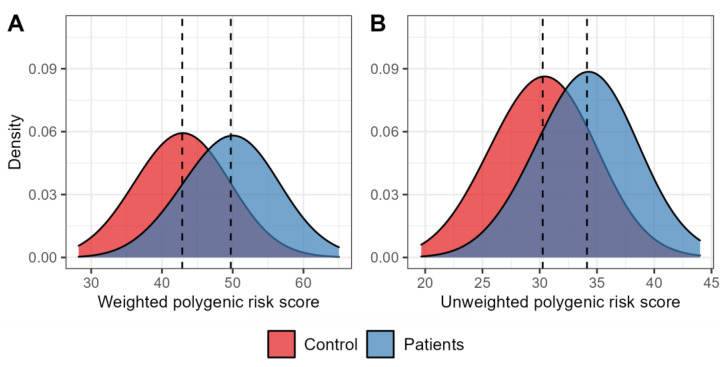
Density plots of polygenic score distributions. (**A**) The plot shows the distribution of weighted risk scores, calculated using odds ratios adjusted for age and sex as weights for the risk alleles of variants significantly linked to metabolic syndrome, with controls represented in red, while individuals with metabolic syndrome are shown in blue. (**B**) This plot depicts the distribution of unweighted polygenic scores, with control participants in red and those with metabolic syndrome in blue. The dotted lines represent mean values for each group.

Figure 2 illustrates the distribution of polygenic scores with and without the locus-specific weights across groups of patients with MetS and healthy controls. The mean values for the weighted and unweighted scores were significantly elevated in MetS patients compared to controls: for the weighted score, 49.73 ± 0.31 vs. 42.87 ± 0.26 (*p* = 8.78 × 10^−53^), and for the unweighted score, 34.13 ± 0.21 vs. 30.26 ± 0.18 (*p* = 3.02 × 10^−39^). The analysis of weighted polygenic scores in relation to MetS revealed that the combined influence of all examined SNVs was linked to a heightened risk of MetS (OR [95%CI_OR_] = 1.28 [1.24–1.34], *p* = 5.73 × 10^−34^). Similarly, the unweighted polygenic score analysis also demonstrated that the collective effect of these polymorphisms was linked to an increased risk of MetS (OR [95%CI_OR_] = 1.37 [1.30–1.45], *p* = 3.51 × 10^−28^).

**Figure 3:**
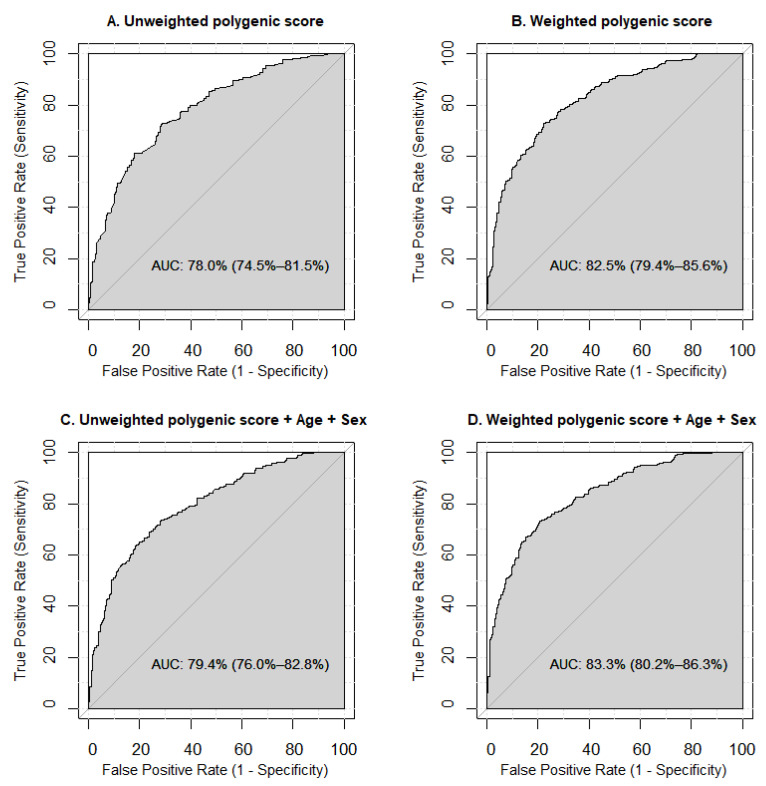
Receiver Operating Characteristic (ROC) curves visualizing the prognostic capabilities of various models in predicting metabolic syndrome. (**A**) The model is based on the unweighted polygenic score derived from the genetic variants that showed significant associations with metabolic syndrome in our study. (**B**) The model is based on the weighted polygenic score, constructed using the odds ratios adjusted for age and sex as weights for the risk alleles of the variants significantly associated with metabolic syndrome in our study. (**C**) The model incorporates the unweighted polygenic score along with age and sex. (**D**) The model incorporates the weighted polygenic score along with age and sex. Sensitivity measures the proportion of true positive results, while specificity measures the proportion of true negative results. The Area Under the Curve (AUC) is used to assess model performance, with AUC values classified as excellent (90% or higher), very good (80–90%), good (70–80%), satisfactory (60–70%), and unsatisfactory (50–60%).

We conducted ROC analysis to evaluate the prognostic accuracy of models built using unweighted and weighted polygenic scores, incorporating additional factors like sex and age (Figure 3). The results showed that the model with the weighted polygenic score outperformed the unweighted polygenic score model (AUC 82.5%, 95%CI 79.4–85.6% vs. AUC 78.0%, 95%CI 74.5–81.5%). Including age and sex as predictors in both the weighted and unweighted polygenic score models slightly improved prediction accuracy (AUC 83.3%, 95%CI 80.2–86.3% for the weighted model, and AUC 79.4%, 95%CI 76.0–82.8% for the unweighted model) (Figure 3).

### 3.3. Net Reclassification Improvement Analysis

Reclassification analysis was performed using net reclassification improvement (NRI) with a reference model that included only non-genetic factors (age and sex) and compared it to models incorporating polygenic scores for the 18 SNVs associated with MetS (listed in Table 1), as well as a model that included all genetic variants studied. We then performed a second NRI analysis, using the model with polygenic scores for the 18 metabolic syndrome-associated variants, age, and sex as the reference. This was compared to the model that included the polygenic risk score for all tested loci, along with age and sex. The results of the NRI analysis are summarized in Table 2. Compared to the model based solely on non-genetic factors, the inclusion of the polygenic score for the MetS-associated variants significantly improved reclassification (NRI = 1.03%, *p* = 3.42 × 10^−50^). However, adding all 40 tested genetic variants did not lead to a significant improvement in predictive power over the model that included the 18 key loci (NRI = −0.11, *p* = 0.507).

## 4. Discussion

We performed an association analysis of polymorphic variants and MetS, as well as phenotypic characteristics of patients with MetS. We evaluated the prognostic ability of a model containing the polygenic risk score for the genetic variants associated with MetS, as identified in our study. Our findings indicate that the most effective prognostic model integrated a weighted polygenic score with non-genetic variables such as age and sex.

The most robust associations with MetS and its endophenotypes in our study were identified for genes involved in β-cell function. Among these, the *CDKAL1* rs9295474 variant exhibited the strongest link to MetS predisposition. This variant has been linked to the development of T2D and hypertension in numerous genome-wide association studies (GWASs) [38,39]. Located on chromosome 6p22.3, the *CDKAL1* gene is closely connected to a heightened risk of T2D and obesity [40]. Expressed predominantly in human pancreatic β-cells, CDKAL1 regulates insulin secretion, sustains β-cell function under glucotoxic conditions, and facilitates the conversion of proinsulin to insulin in response to glucose stimulation [41]. Moreover, it plays a critical role in maintaining mitochondrial morphology and adipose tissue function [40,42]. The progressive dysfunction of pancreatic β-cells exacerbates MetS and its complications, establishing a pathogenic feedback loop. Supporting this, recent studies have shown that *CDKAL1* variants are associated with a greater waist circumference and WHR in Chinese populations [43] and serve as independent predictors of metabolically healthy obesity in Chinese children [44].

Similarly, *ADCY3* rs17799872 demonstrated associations in our study with a broad spectrum of traits, encompassing anthropometric (BMI, height, waist circumference, hip circumference, WHR), glycemic (IFG, HOMA-IR, fasting and postprandial insulin), and inflammatory markers (ESR, CRP, fibrinogen, TNF-α). Prior research has linked SNPs in the *ADCY3* gene to several of these traits, including waist circumference [45], WHR [46], whole body fat mass [47], BMI (adjusted for smoking behavior) [48], MetS [3], FG, and SBP [49]. The *ADCY3* gene encodes adenylyl cyclase 3, a key enzyme located in the hypothalamus that governs multiple critical pathways. Mutations in *ADCY3* have been identified in children with severe monogenic obesity [50], while polygenic variants increase the risk of obesity and T2D, with the effect being more pronounced in homozygous carriers [51]. Rare *ADCY3* mutations have been associated with impaired appetite control, contributing to early-onset severe obesity and insulin resistance [52].

Neurotransmitters emerged as another gene cluster strongly associated with MetS in our analysis. Notably, *ADRA2A* rs1800544 was linked to HOMA-IR and postprandial insulin levels, consistent with earlier findings that associated this variant with MetS and obesity [8,53]. The *ADRA2A* gene regulates catecholamine function, which plays a pivotal role in energy consumption and lipolysis—the hydrolysis of stored triglycerides into free fatty acids and glycerol. In adipocytes, β-adrenergic receptors (ADRB) stimulate lipolysis, while α2-adrenergic receptors (ADRA2) suppress it. Additionally, insulin acts as a key suppressor of catecholamine-driven lipolysis, underscoring the complex interplay of these pathways in metabolic regulation [53].

Another significant finding was the strong association between *NPY2R* rs1047214 and MetS. The *NPY2R* gene encodes a receptor for neuropeptide Y, an orexigenic agent whose production is modulated by blood glucose levels. *NPY2R* polymorphisms have been associated with BMI and show gender-specific effects; for instance, certain SNVs in this gene appear to influence obesity risk exclusively in men [54]. Interestingly, related genetic variants in *PYY* (encoding the ligand for NPY2R) have been associated with obesity-related traits solely in women [55]. These findings suggest that appetite regulation pathways exhibit gender-specific effects on body composition, although the underlying mechanisms remain insufficiently understood.

SNVs in muscarinic acetylcholine receptor genes *CHRM1* and *CHRM4* were also associated with MetS in our study. Muscarinic acetylcholine receptors, part of the G-protein-coupled receptor family, mediate the diverse cellular effects of acetylcholine in both the central and peripheral nervous systems. These effects include the inhibition of adenylate cyclase, degradation of phosphoinositides, and mediation of potassium channel activity [56,57]. *CHRM1* rs2067477 has previously been linked to WHR adjusted for BMI [58] and the waist-to-hip index [59], while *CHRM4* rs2067482, although not previously associated with metabolic conditions, has been implicated in headache or migraine [60], postoperative delirium, and postoperative cognitive dysfunction [56], as well as schizophrenia [61].

SNVs in genes related to adiposity were among those associated with MetS in our study. *SEC16B* rs10913469, previously linked to MetS [62], BMI [63], and waist circumference [64], has also been implicated in childhood obesity, with associations influenced by gender, age, and nutritional status [65]. Similarly, *GHRL* rs696217 has been tied to obesity and HDL cholesterol levels [66,67]. The *GHRL* gene encodes preproghrelin, a precursor hormone processed into ghrelin and obestatin, predominantly secreted by stomach cells. Ghrelin, a key player in the orexigenic signaling system, is also associated with addictive behaviors, including alcohol use disorder, compulsive overeating [68,69,70], and drug addiction [71]. In rodents, ghrelin activates cholinergic–dopaminergic reward pathways [72]. Another notable variant, *FTO* rs9939609, has consistently demonstrated associations with MetS across studies [73,74,75]. This SNV exhibits pleiotropic effects, influencing various glycemic, cardiovascular, lipid, and anthropometric traits while also affecting the age of onset for conditions like diabetes, cancer, cardiovascular disease, and neurodegenerative disorders [76].

SIRT1, a member of the sirtuin family of NAD^+^-dependent enzymes responsible for histone deacetylation, is a key regulator of apoptosis and is essential for glucose and lipid metabolism. The genetic variant *SIRT1* rs3818292 has been linked to obesity [77] and MetS [78]. Extensive research has highlighted the significance of SIRT1 in maintaining glucose homeostasis and supporting cardiovascular health [79]. In diabetic animal models, *SIRT1* expression in islet β-cells was shown to stimulate insulin release triggered by glucose intake [80]. Furthermore, studies in prediabetic individuals revealed an association between insulin resistance and reduced *SIRT1* expression in subcutaneous fat, accompanied by elevated serum levels of inflammatory cytokines. These findings suggest that SIRT1 activity in adipose tissue may confer cardioprotective benefits, particularly in prediabetic conditions [81].

As evidenced by our findings, overlapping pathways in MetS involve key genes that influence various aspects of the condition. Insulin resistance, a core feature of MetS, is linked to genes such as *CDKAL1*, *ADCY3*, and *ADRA2A*. Obesity and adiposity are influenced by genes like *ADCY3*, *NPY2R*, and *SEC16B*, which play central roles in MetS pathophysiology. Chronic inflammation, a critical factor in MetS progression, is associated with inflammatory markers tied to genes such as *ADCY3* and *SIRT1*. Additionally, variants in genes like *NPY2R*, *ADRA2A*, and *GHRL* emphasize the role of energy homeostasis and appetite regulation in MetS risk. These overlapping pathways highlight the complexity of MetS, with interactions between insulin resistance, obesity, inflammation, and hormonal regulation, where genetic variants contribute to individual risk profiles.

We developed a polygenic score using genetic variants associated with MetS and evaluated the prognostic performance of models incorporating both unweighted and weighted polygenic scores, along with age and sex, for predicting MetS risk. Our findings revealed that the most predictive model combined a weighted polygenic score with non-genetic factors such as sex and age. Previously, Park et al. constructed a three-factor model, which groups MetS components into latent factors labeled as obesity, insulin resistance/hypertension, and dyslipidemia, which demonstrated an excellent model fit based on metrics like the comparative fit index (CFI, 0.981–0.996) and standardized root mean square residual (SRMR, 0.036–0.043) [3]. The model effectively captured the clustering of MetS components, and the further constructed hierarchical factor model integrated shared genetic effects among these latent factors, highlighting the interrelated nature of MetS [3]. However, this approach primarily provides structural insights into the relationships among MetS components rather than directly evaluating predictive power in new individuals. Van Walree et al. employed the MetS factor GWAS summary statistics to calculate the polygenic score that explained 5.9% of the variance in MetS, which is higher than the variance explained by the individual MetS component polygenic score [82]. When combined with covariates, the polygenic risk model explained 21% of the variance (Nagelkerke R^2^) [82]. While statistically significant (*p* = 0.0058) and demonstrating better predictive abilities than the sum of its parts, the polygenic risk-based model captured a relatively modest proportion of the variance [82].

Prior studies have demonstrated that combining polygenic scores with other risk factors such as demographic information, lifestyle choices, medication use, and comorbidities can enhance risk stratification [83]. Population-specific genetic structures, including patterns of linkage disequilibrium, may affect the influence of the studied variants on MetS and the varying prognostic values of weighted and unweighted polygenic scores. Multiethnic polygenic scores have shown superior performance compared to those developed primarily in European populations for predicting health risks [84]. Moreover, incorporating key SNVs along with demographic factors including sex and ethnicity has been found to enhance the accuracy of polygenic scores [85]. Although increasing the number of genetic variants used to calculate polygenic scores can potentially improve prognostic power [86], our findings suggest that including all tested SNVs did not substantially enhance the model’s prognostic ability (Table 2). Validation in an independent cohort will be necessary to confirm the constructed model’s predictive capacity.

### Study Strengths and Limitations

Our study provides valuable insights into the genetic basis of MetS by focusing on key genes related to adiposity, β-cell function, inflammation, and neurotransmitter activity. The inclusion of a polygenic score combining genetic and non-genetic factors, such as age and sex, enhances our understanding of MetS risk prediction. These insights into genetic associations and polygenic scores hold potential for clinical applications, including the development of diagnostic tools and the identification of targets for drug development.

However, several limitations must be acknowledged. The sample size, while adequate for detecting significant associations, may have limited our ability to identify smaller-effect variants. Additionally, while we focused on 40 key genes, it is possible that other variants outside these genes may also contribute to MetS susceptibility. These factors underscore the need for replication in larger, independent cohorts to validate our findings and extend their applicability across different populations.

## 5. Conclusions

Our analysis identified several key genetic variants associated with MetS and its endophenotypes, particularly those involved in β-cell function and metabolic regulation. Notably, variants in the *CDKAL1*, *ADCY3*, *ADRA2A*, and *NPY2R* genes showed strong associations with metabolic traits such as waist circumference, insulin resistance, and obesity. These findings contribute to our understanding of the genetic underpinnings of MetS, emphasizing the role of genes regulating insulin secretion, appetite, and fat metabolism. Our study highlights the potential of polygenic scores in predicting MetS risk, with the most predictive model incorporating weighted polygenic scores along with age and sex as non-genetic factors. Although expanding the polygenic scores to include additional variants did not significantly improve model performance, our results suggest that integrating genetic and demographic data could enhance the accuracy of MetS risk prediction. Further validation in diverse populations is essential to confirm the robustness of these models and their applicability in clinical settings.

## Figures and Tables

**Table 1:** Loci significantly associated with metabolic syndrome.

Gene	SNV	EA ^1^	MA ^2^	MAF ^3^		P_HWE_ ^4^	OR ^5^ (95%CI_OR_) ^6^	P ^7^	P_FDR_ ^8^
				Control	MetS				
*TNFRSF1B*	rs1061624	A	G	0.51	0.47	0.088	1.32 (1.04–1.69)	0.022	0.049
*LEPR*	rs1137100	G	G	0.21	0.29	0.294	1.53 (1.17–2.01)	0.002	0.007
*CRP*	rs2794521	C	C	0.21	0.29	0.546	1.51 (1.16–1.97)	0.002	0.007
*SEC16B*	rs10913469	T	C	0.23	0.17	0.197	1.76 (1.3–2.38)	2.29 × 10^−4^	1.14 × 10^−3^
*ADCY3*	rs17799872	A	A	0.08	0.13	0.498	1.57 (1.08–2.29)	0.018	0.041
*GHRL*	rs696217	A	A	0.08	0.14	0.479	1.89 (1.33–2.68)	3.67 × 10^−4^	1.63 × 10^−3^
*NPY2R*	rs1047214	C	T	0.52	0.38	0.067	2.12 (1.64–2.74)	7.47 × 10^−9^	1.50 × 10^−7^
*CDKAL1*	rs9295474	C	G	0.36	0.16	0.827	2.63 (1.97–3.5)	4.47 × 10^−11^	1.79 × 10^−9^
*LPL*	rs295	C	C	0.25	0.35	0.789	1.46 (1.16–1.85)	0.002	0.006
*SIRT1*	rs3818292	G	G	0.21	0.28	0.364	1.39 (1.07–1.81)	0.014	0.034
*ADRA2A*	rs1800544	G	G	0.14	0.25	0.145	2.18 (1.62–2.95)	2.98 × 10^−7^	3.98 × 10^−6^
*BDNF*	rs11030107	G	G	0.13	0.23	0.515	1.9 (1.38–2.61)	7.49 × 10^−5^	4.99 × 10^−4^
*CHRM4*	rs2067482	C	T	0.08	0.04	1	2.29 (1.34–3.9)	0.002	0.007
*CHRM1*	rs2067477	A	A	0.04	0.12	1	3.08 (1.91–4.97)	3.77 × 10^−6^	3.01 × 10^−5^
*HTR3A*	rs1062613	T	T	0.19	0.3	0.068	2.01 (1.5–2.69)	2.30 × 10^−6^	2.30 × 10^−5^
*ZBTB42*	rs3803300	A	A	0.03	0.07	1	2.34 (1.32–4.16)	0.004	0.010
*FTO*	rs9939609	A	A	0.18	0.27	0.607	1.72 (1.3–2.27)	1.52 × 10^−4^	8.66 × 10^−4^
*GIPR*	rs2302382	C	A	0.23	0.16	0.391	1.55 (1.16–2.08)	0.003	0.010

^1^ EA—effect allele; ^2^ MA—minor allele; ^3^ MAF—minor allele frequency; ^4^ P_HWE_—significance level for the Hardy–Weinberg test; ^5^ OR—odds ratio; ^6^ 95%CI_OR_—95% confidence interval for the odds ratio; ^7^ P—significance level; ^8^ P_FDR_—significance level after Benjamani–Hochberg adjustment.

**Table 2:** Comparison of net reclassification improvement between models with added parameters and the reference.

	Reference (Age + Sex)				Reference (18SNV Polygenic Score +Age + Sex)			
	18SNV ^1^ Polygenic Score + Sex + Age				40SNV Polygenic Score + Sex + Age			
	NRI ^2^	SE ^3^	95%CI ^4^	*p*-Value ^5^	NRI	SE	95%CI	*p*-Value
Total	1.03	0.07	(0.89, 1.16)	3.42 × 10^−50^	−0.11	0.16	(−0.42, 0.23)	0.507
Cases	0.55	0.04	(0.45, 0.61)	1.45 × 10^−40^	−0.08	0.08	(−0.25, 0.07)	0.363
Controls	0.48	0.04	(0.41, 0.57)	7.52 × 10^−34^	−0.03	0.09	(−0.19, 0.18)	0.718

^1^ SNV—single nucleotide variant; ^2^ NRI—net reclassification improvement; ^3^ SE—standard error; ^4^ 95%CI—95% confidence interval; ^5^ *p*-value—significance level; NRI values are expressed as percentages.

## Data Availability

Data are contained within the article and Supplementary Materials.

## Supplementary Materials

The following supporting information can be downloaded at: https://www.mdpi.com/article/10.3390/genes16010022/s1, Figure S1: Genome-wide association study results for phenotypic traits for the analyzed genetic variants; Table S1: Associations between studied loci and metabolic syndrome; Table S2: Associations between studied loci and metabolic syndrome endophenotypes.
