# Supplementary material for: Polygenic Score Approach to Predicting Risk of Metabolic Syndrome
Source: Genes (Basel). 2024 Dec 26;16(1):22. doi: 10.3390/genes16010022 (PMC11764775; doi:10.3390/genes16010022)
Supplement: Supplementary file 1 [file genes-16-00022-s001.zip › Supplementary_Figures.pdf]

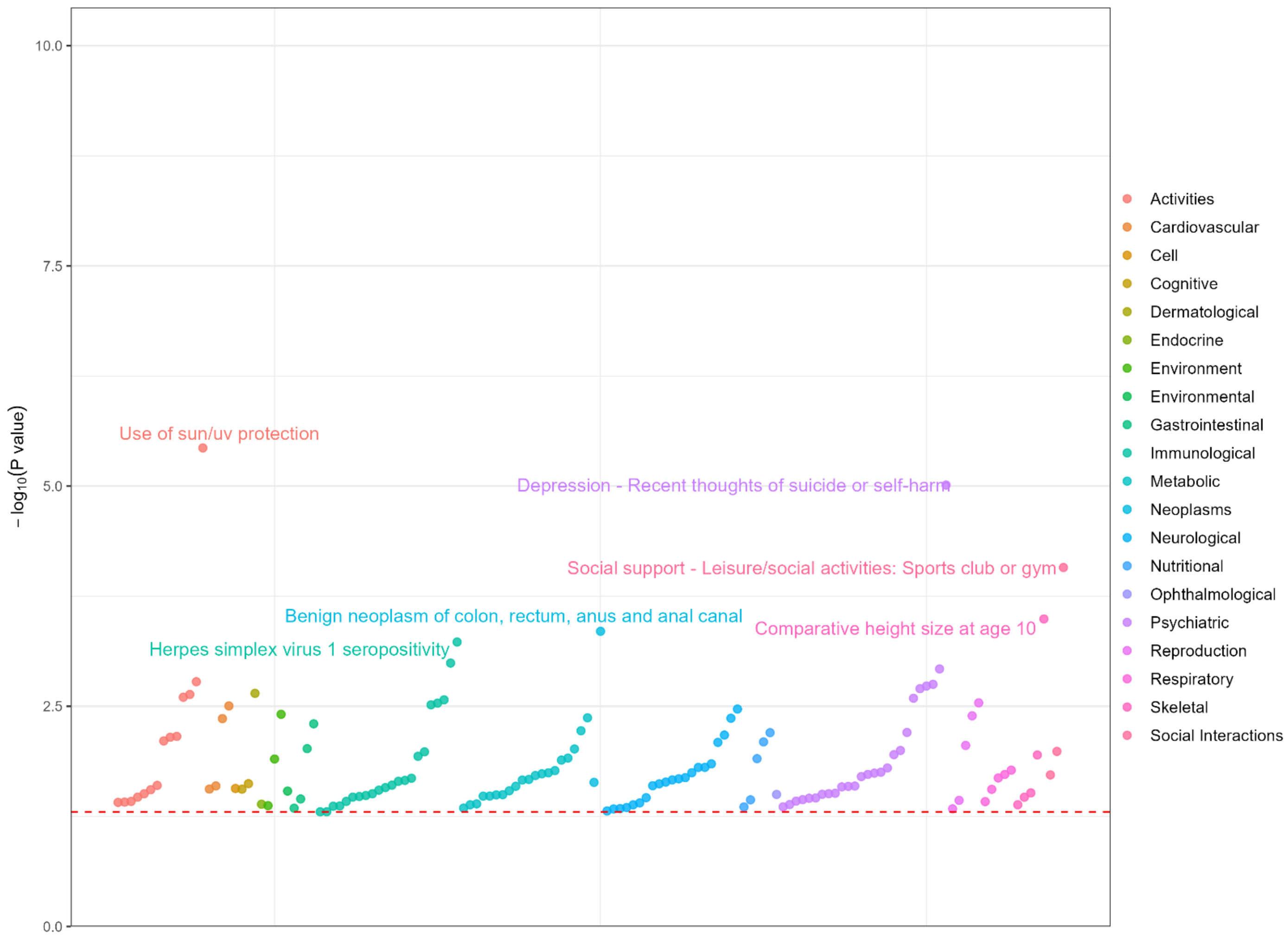

The results of the genome-wide association studies with phenotypic traits for the *TNFRSF1B rs1061624* variant

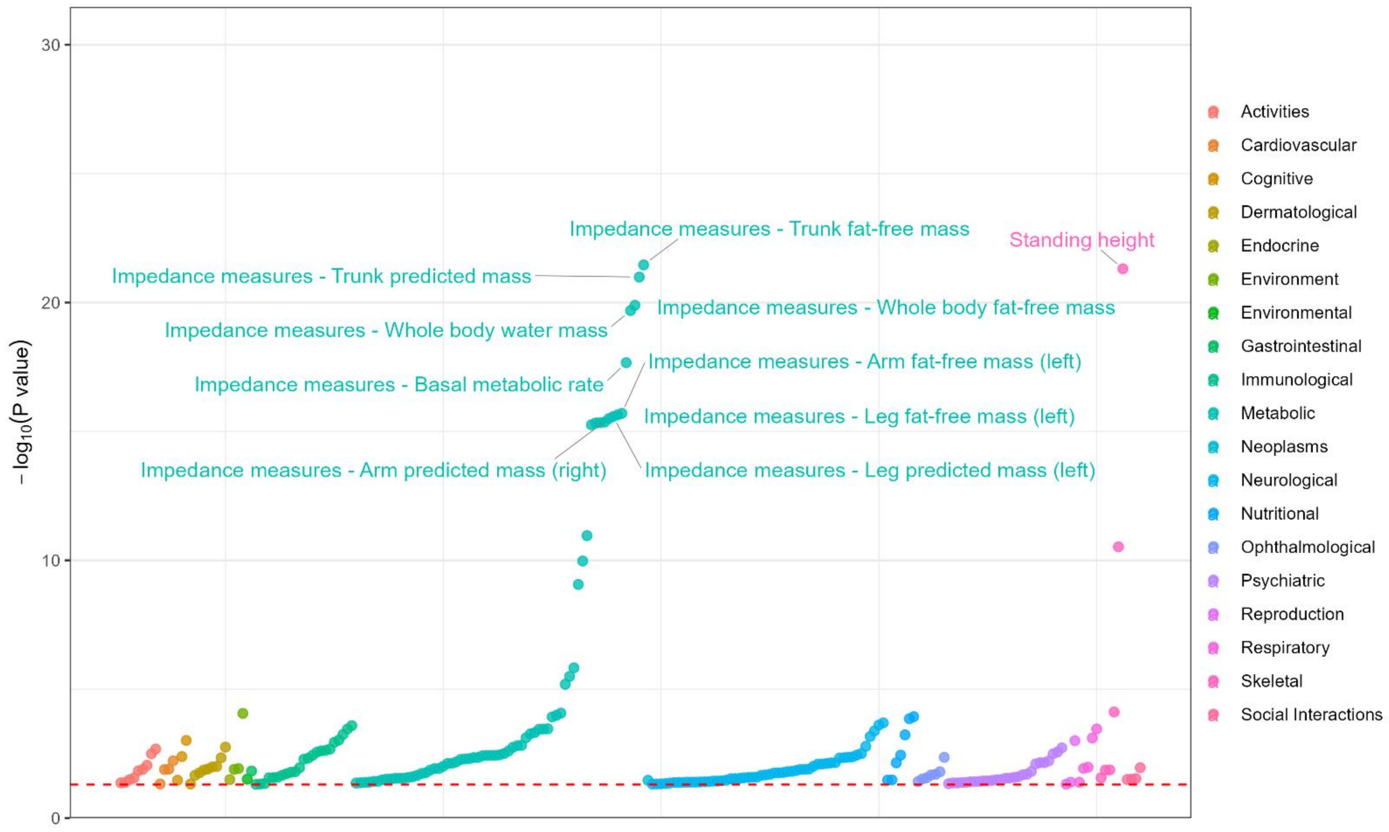

The results of the genome-wide association studies with phenotypic traits for the *HTR1D rs623988* variant

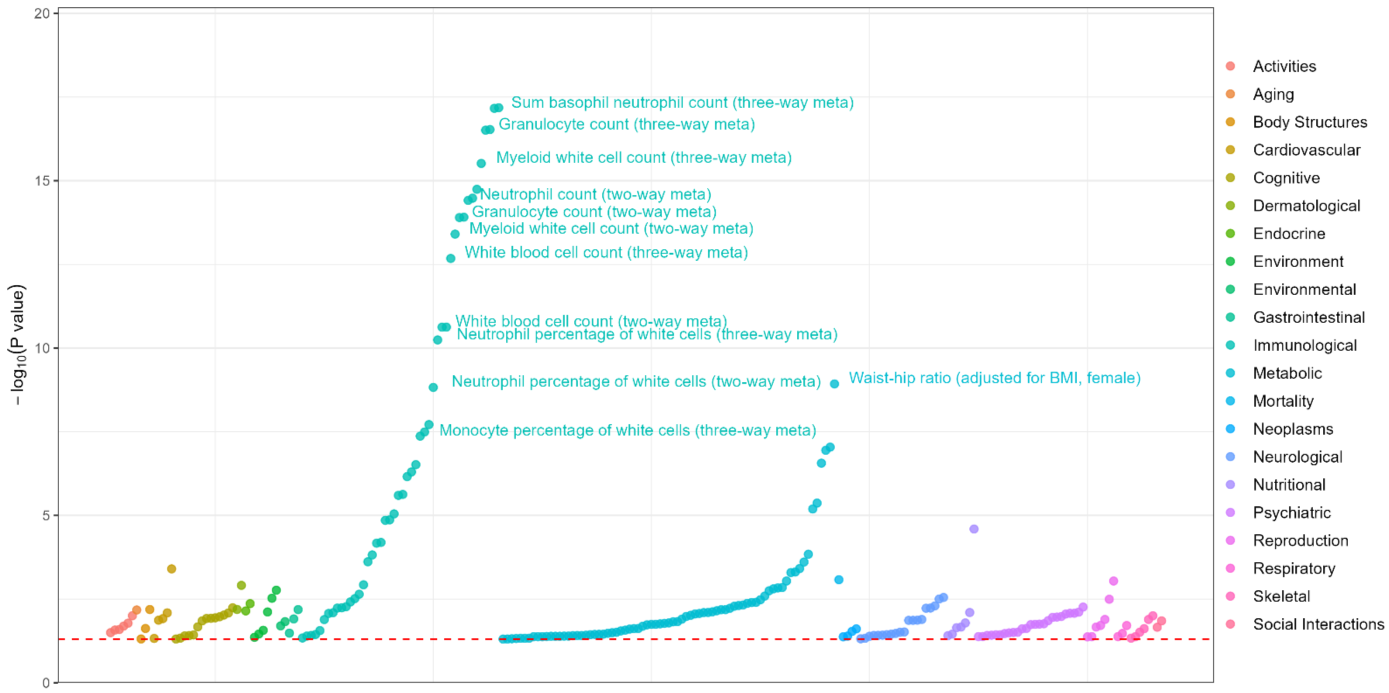

The results of the genome-wide association studies with phenotypic traits for the *LEPR rs1137100* variant

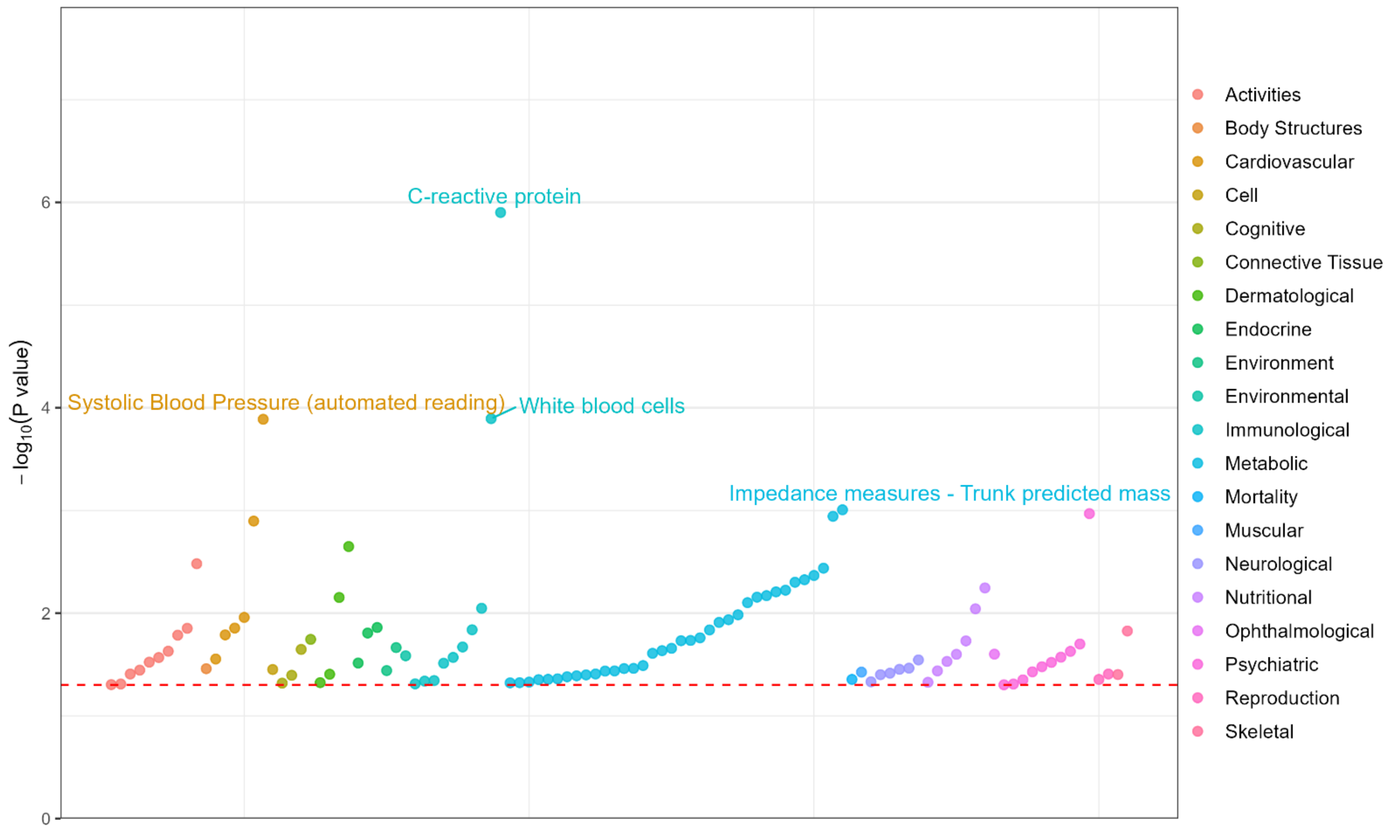

The results of the genome-wide association studies with phenotypic traits for the *CRP rs2794521* variant

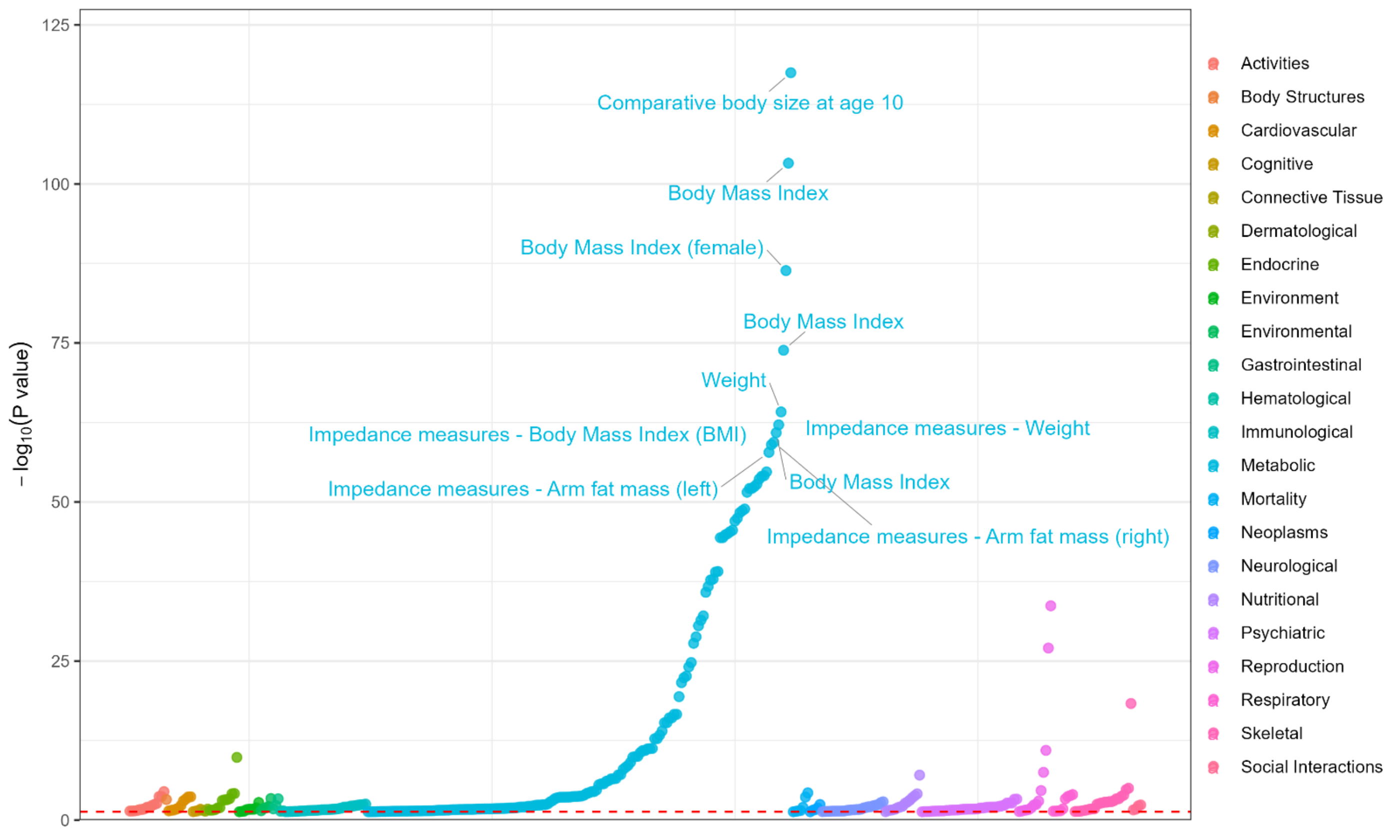

The results of the genome-wide association studies with phenotypic traits for the *SEC16B rs10913469* variant

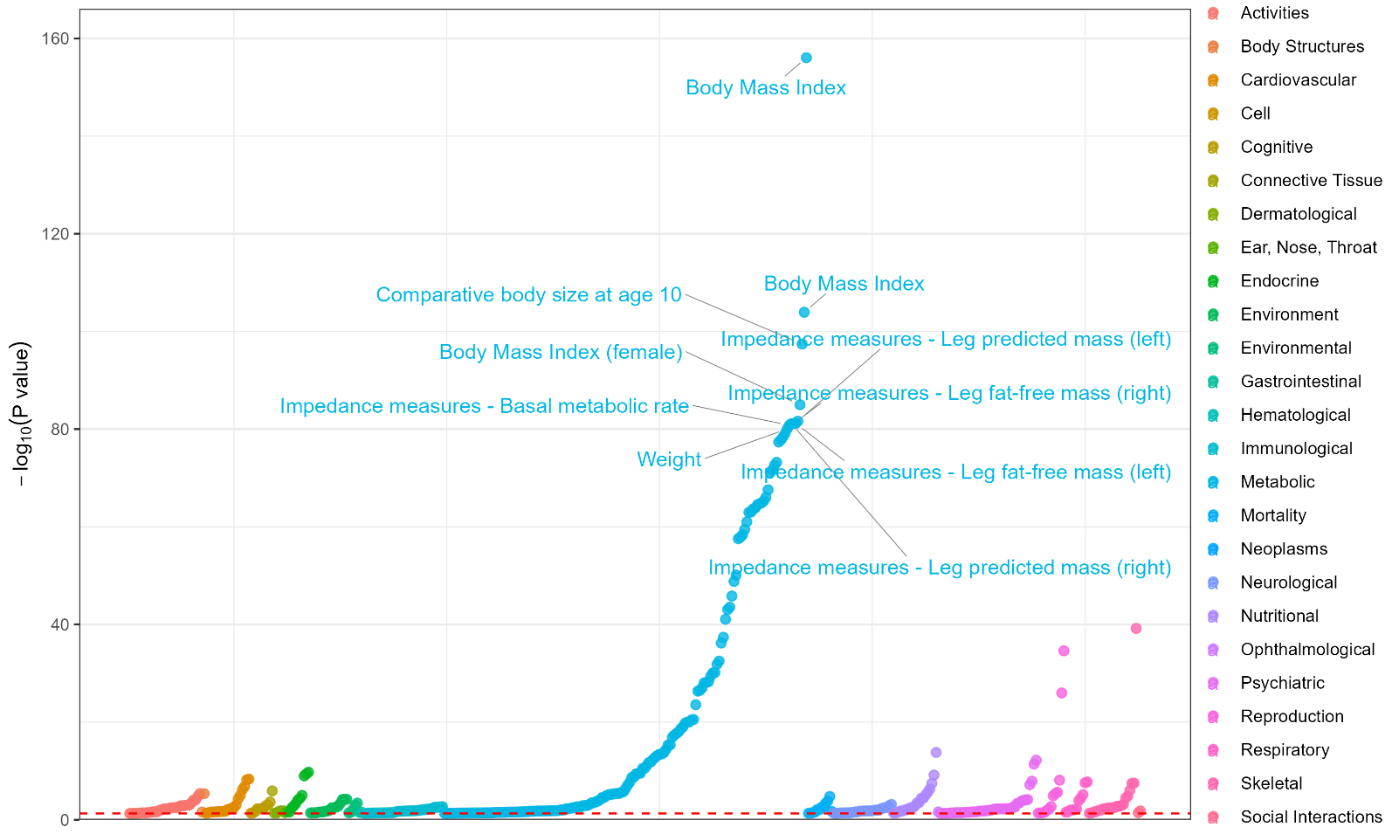

The results of the genome-wide association studies with phenotypic traits for the *TMEM18 rs6548238* variant

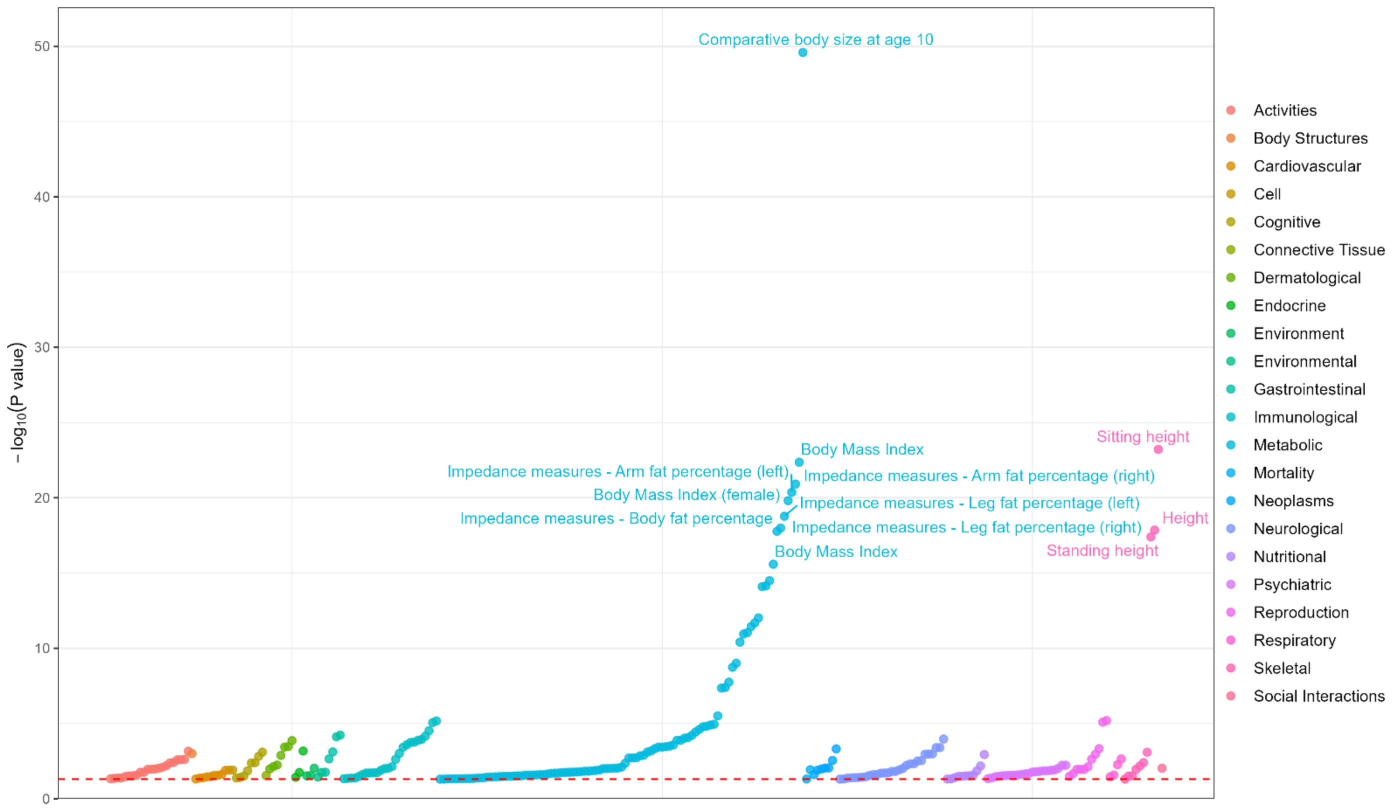

The results of the genome-wide association studies with phenotypic traits for the *ADCY3 rs17799872* variant

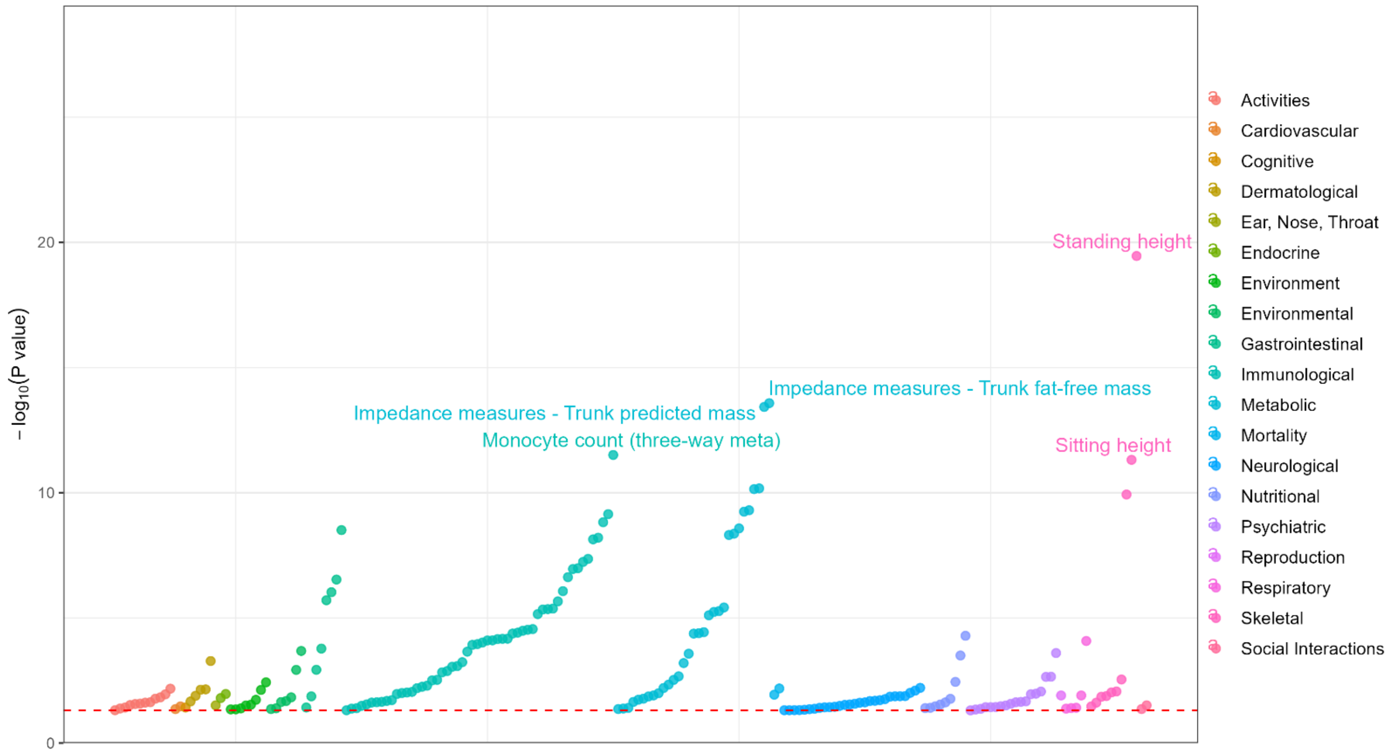

The results of the genome-wide association studies with phenotypic traits for the *CXCR2 rs2230054* variant

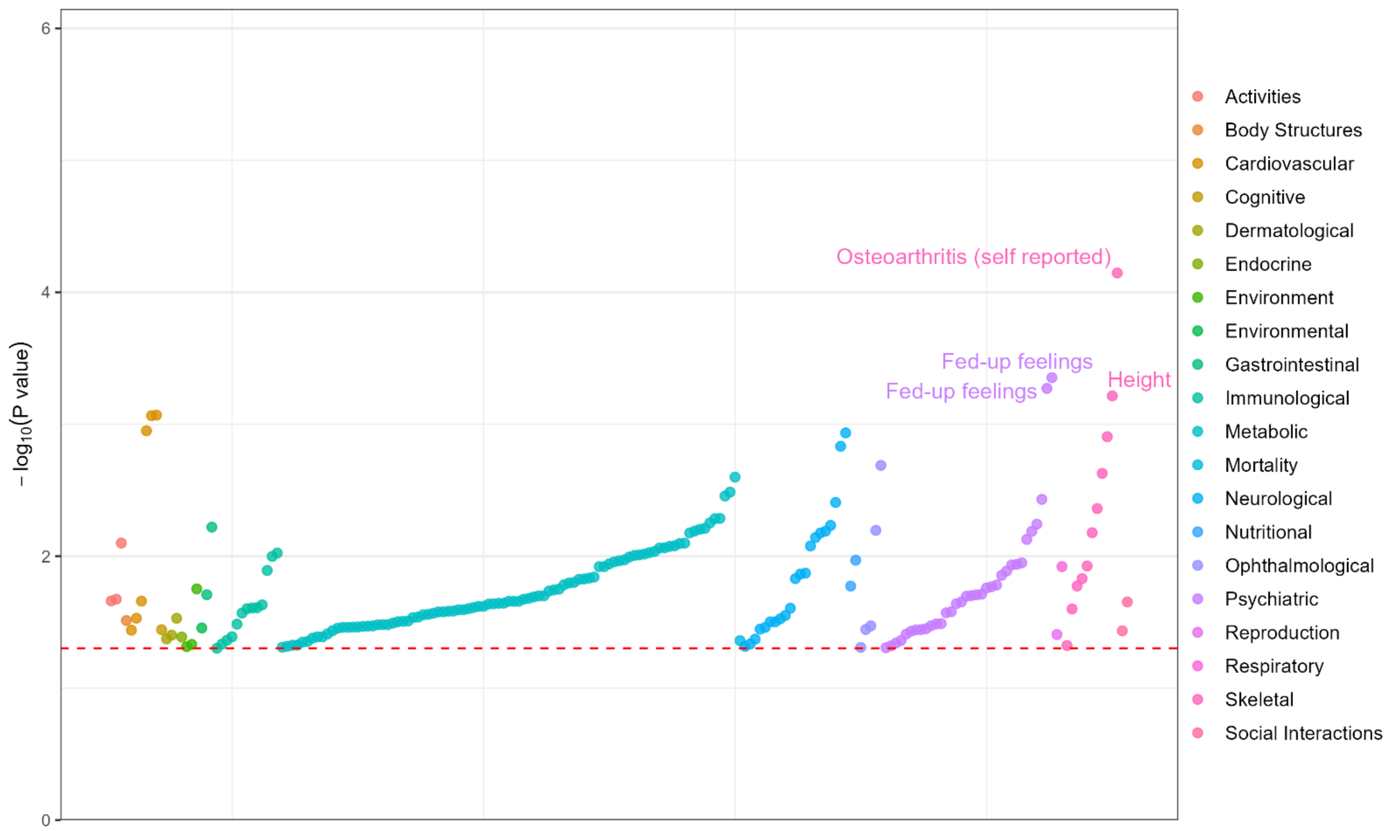

The results of the genome-wide association studies with phenotypic traits for the *GHRL rs696217* variant

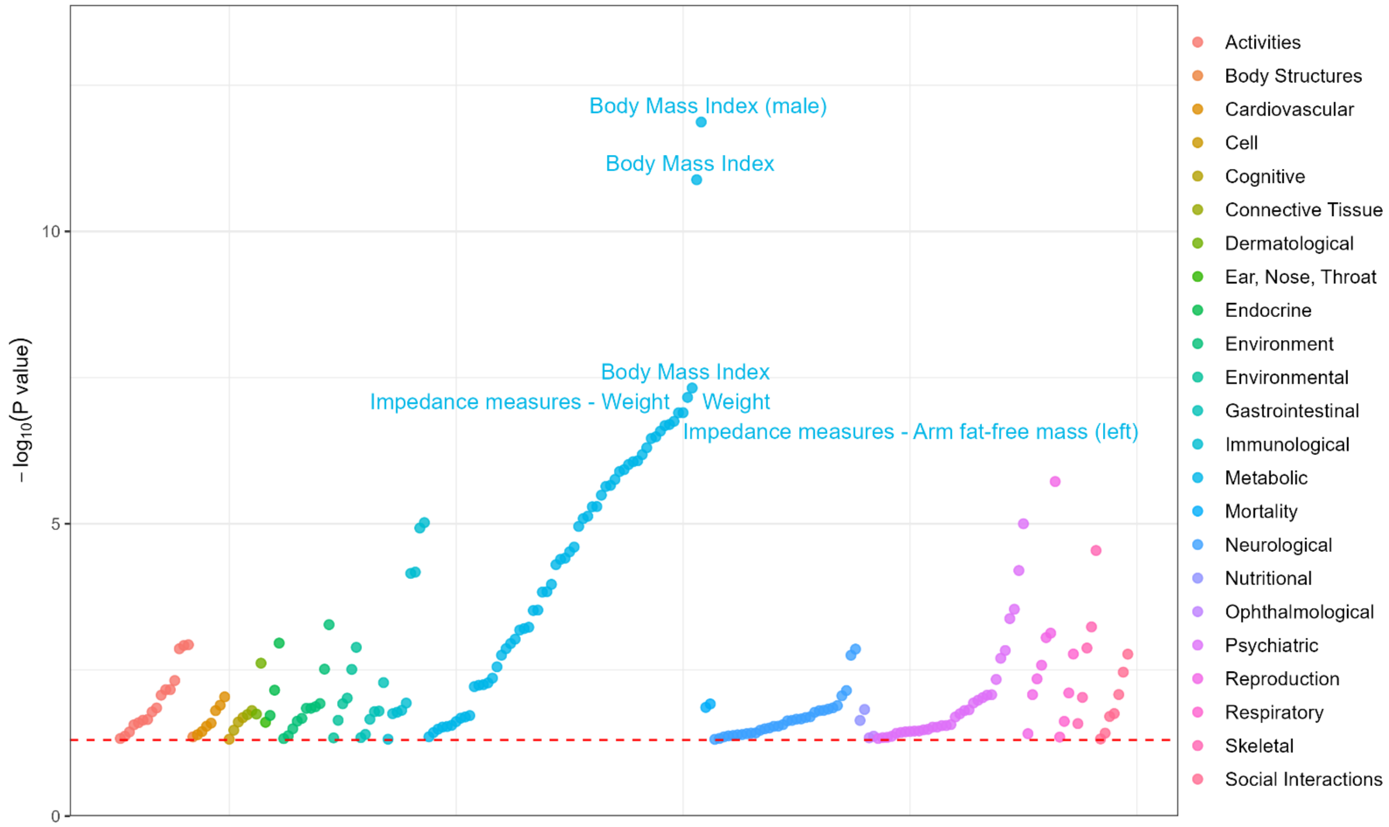

The results of the genome-wide association studies with phenotypic traits for the *HTR1F rs56398417* variant

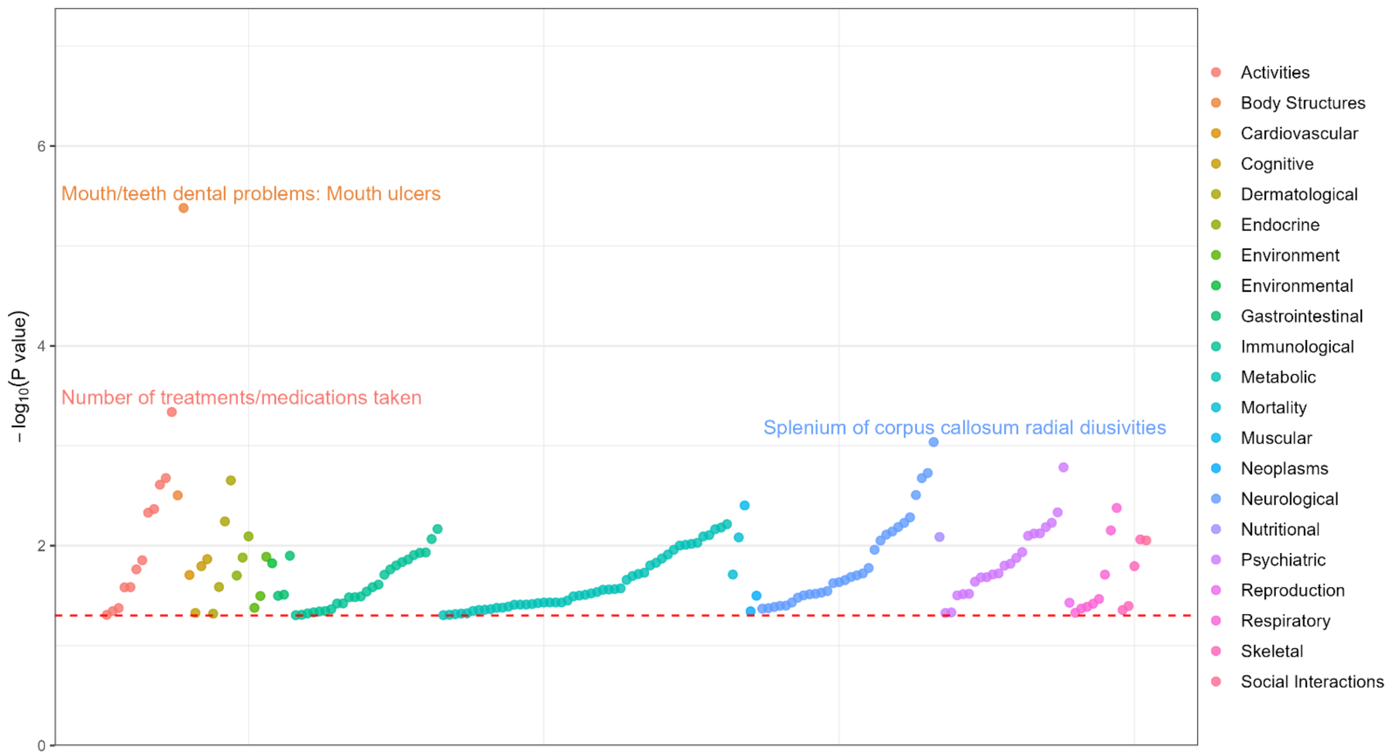

The results of the genome-wide association studies with phenotypic traits for the *IL12A rs2243115* variant

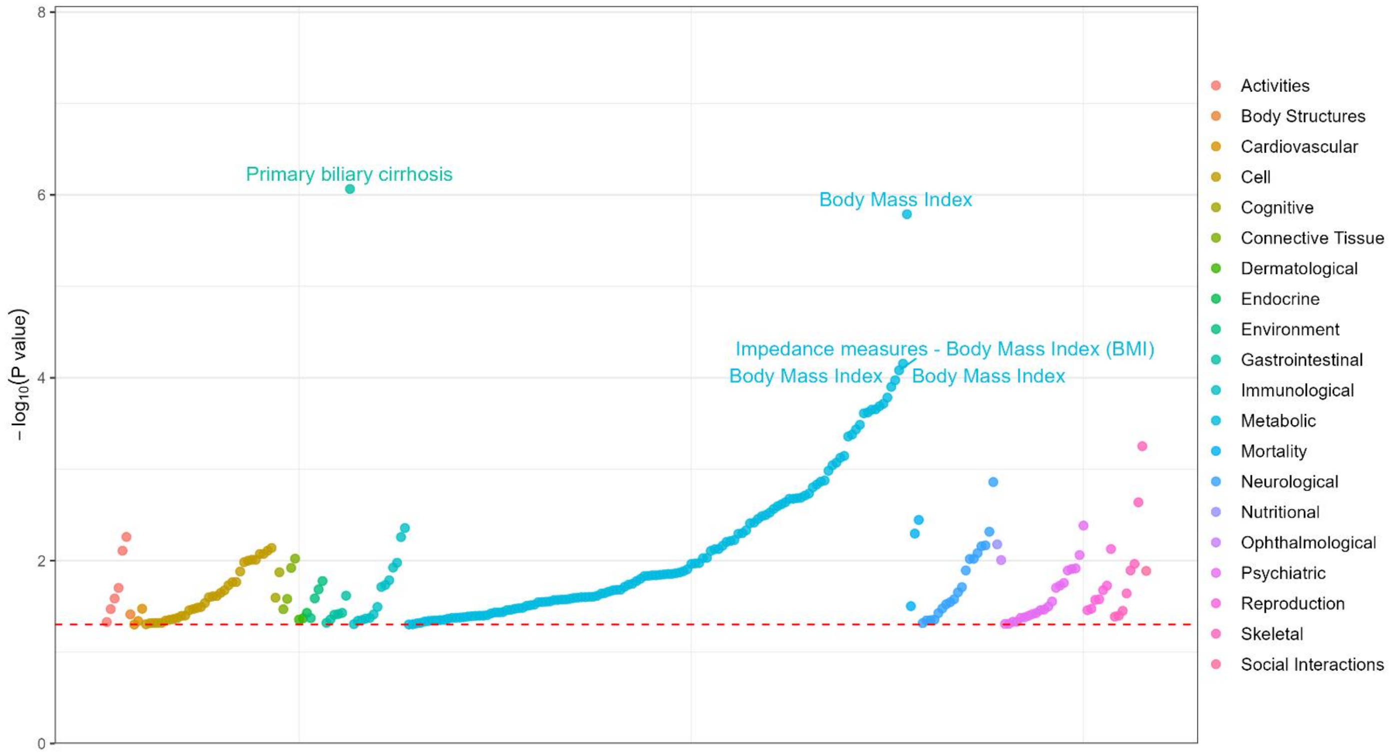

The results of the genome-wide association studies with phenotypic traits for the *IL12A rs568408* variant

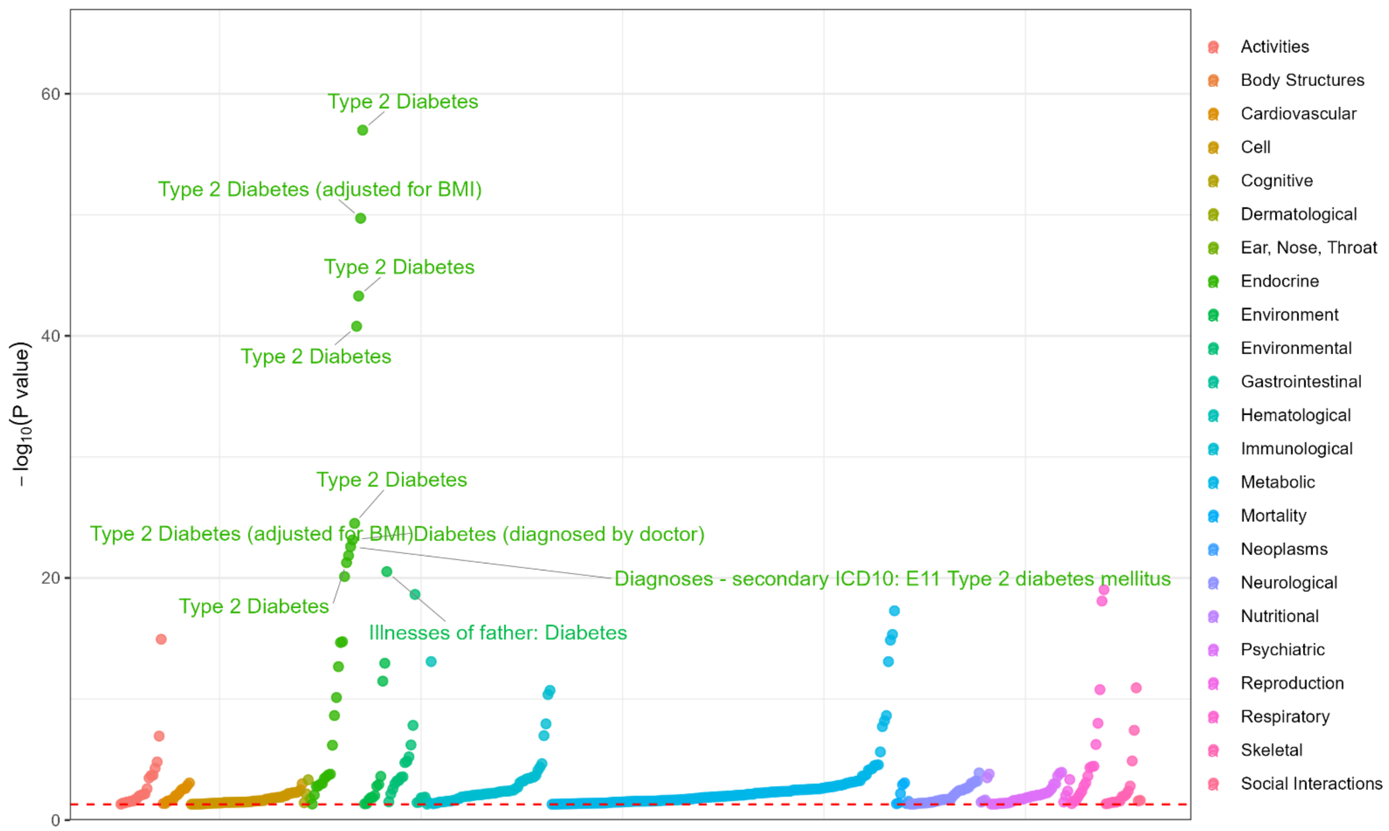

The results of the genome-wide association studies with phenotypic traits for the *IGF2BP2 rs1470579* variant

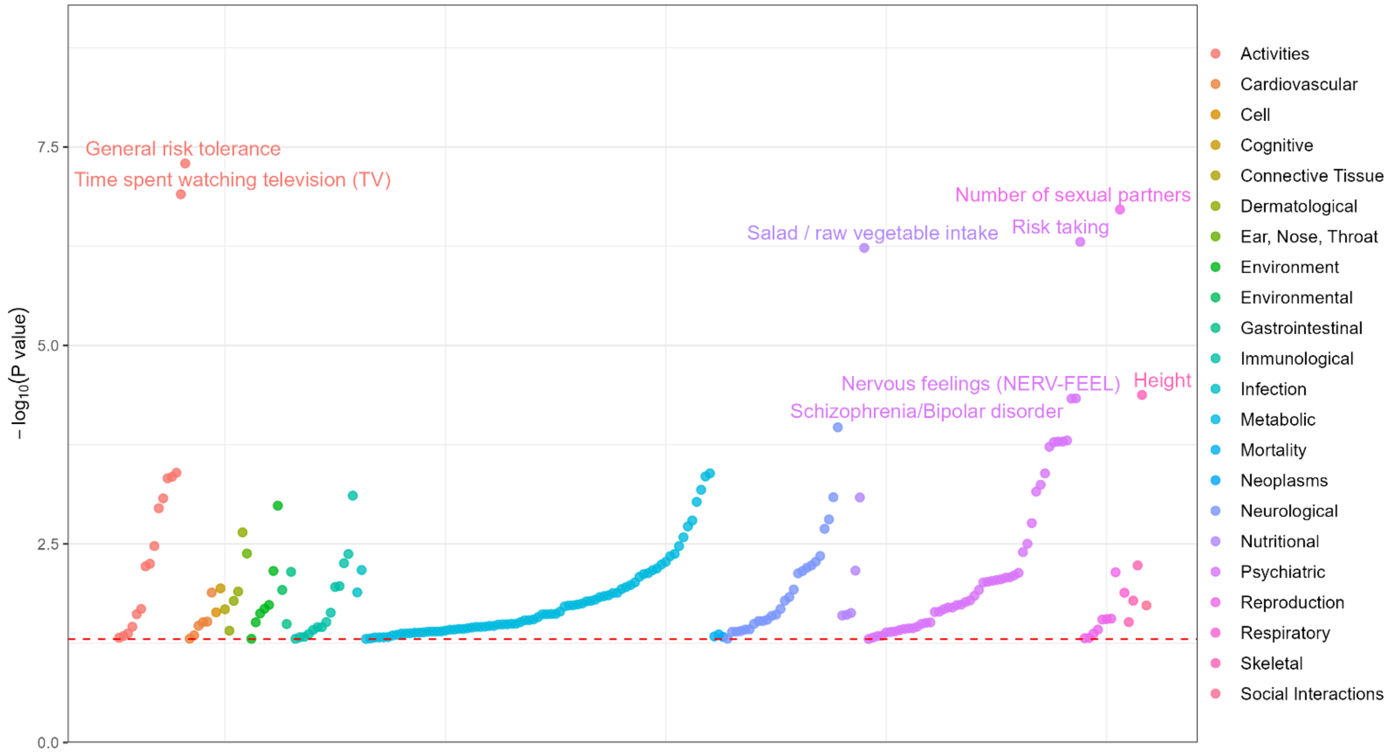

The results of the genome-wide association studies with phenotypic traits for the *GABRA2 rs279845* variant

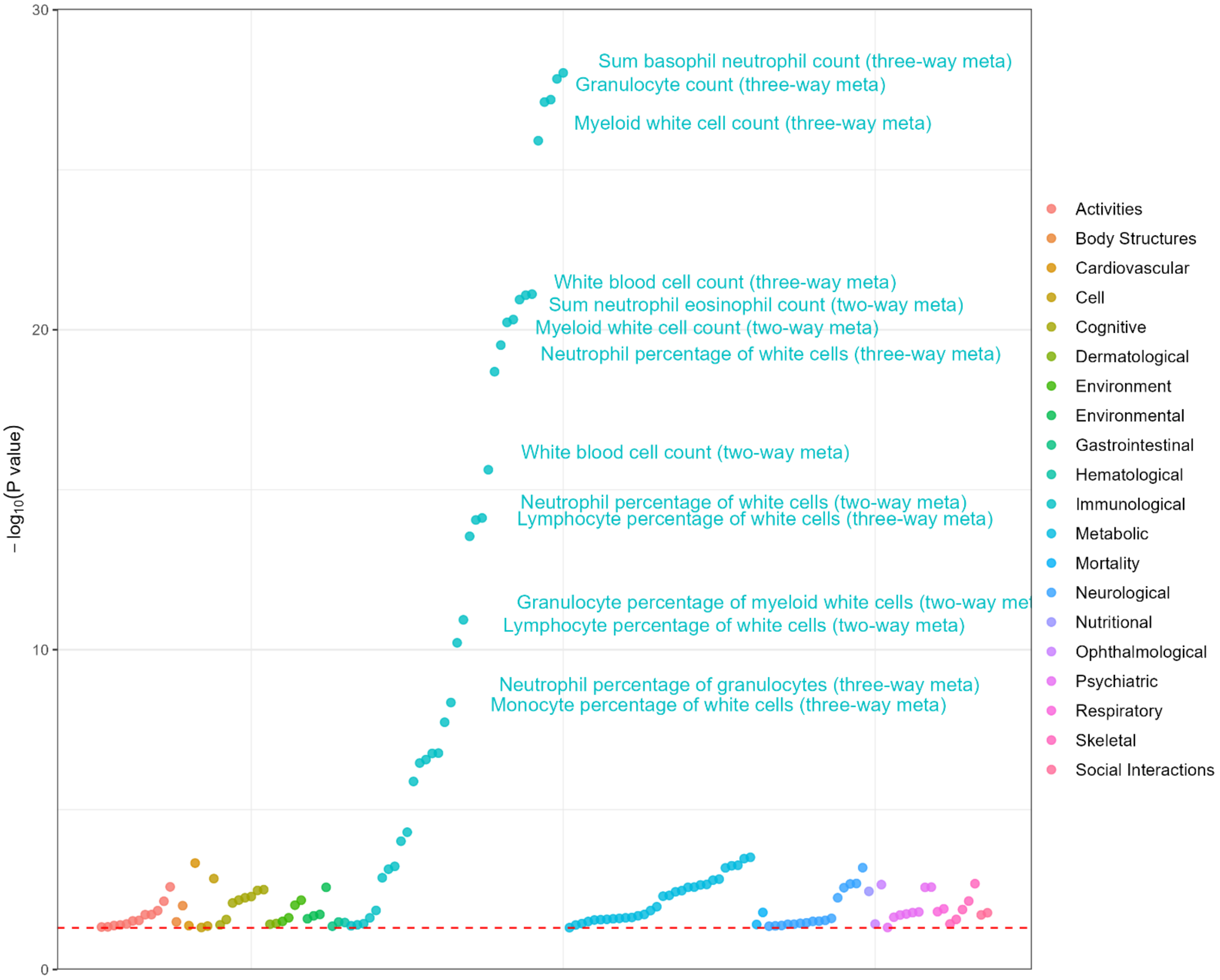

The results of the genome-wide association studies with phenotypic traits for the *CXCL8 rs4073* variant

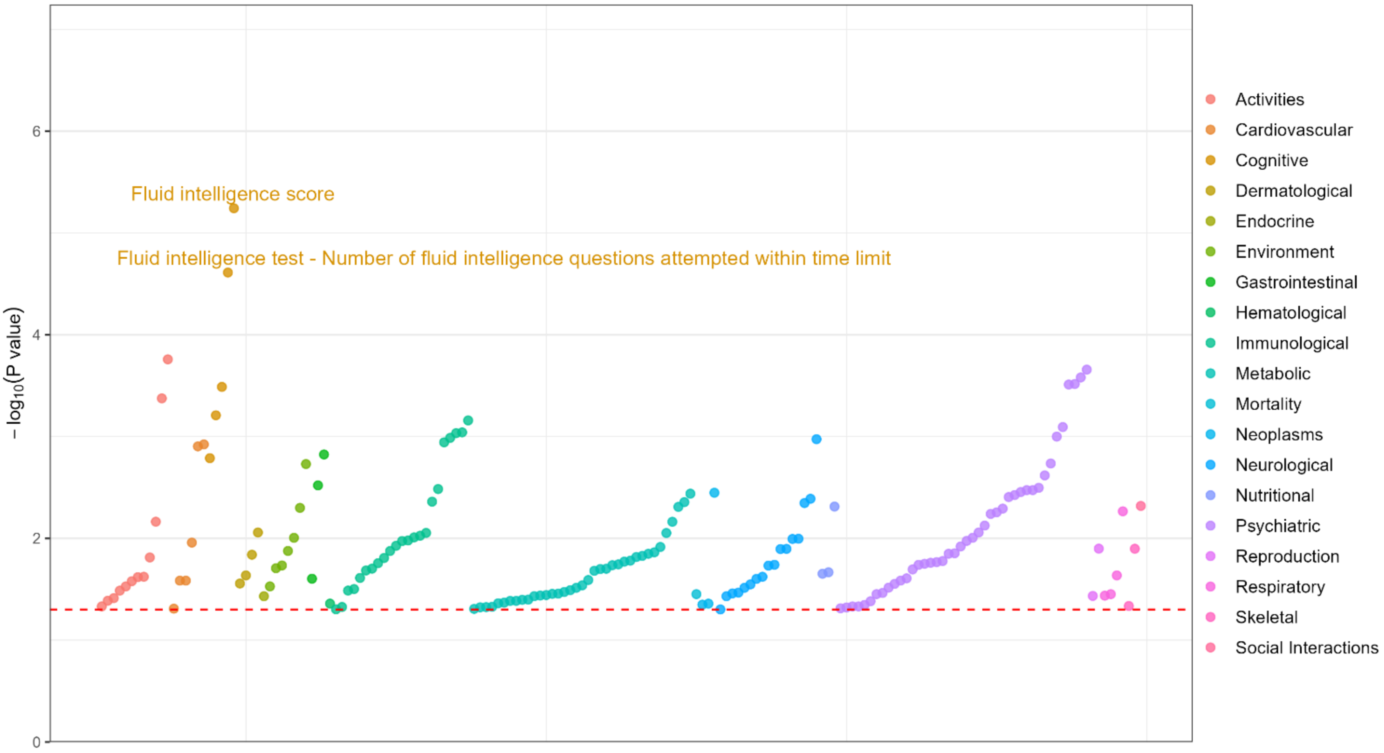

The results of the genome-wide association studies with phenotypic traits for the *NPY2R rs1047214* variant

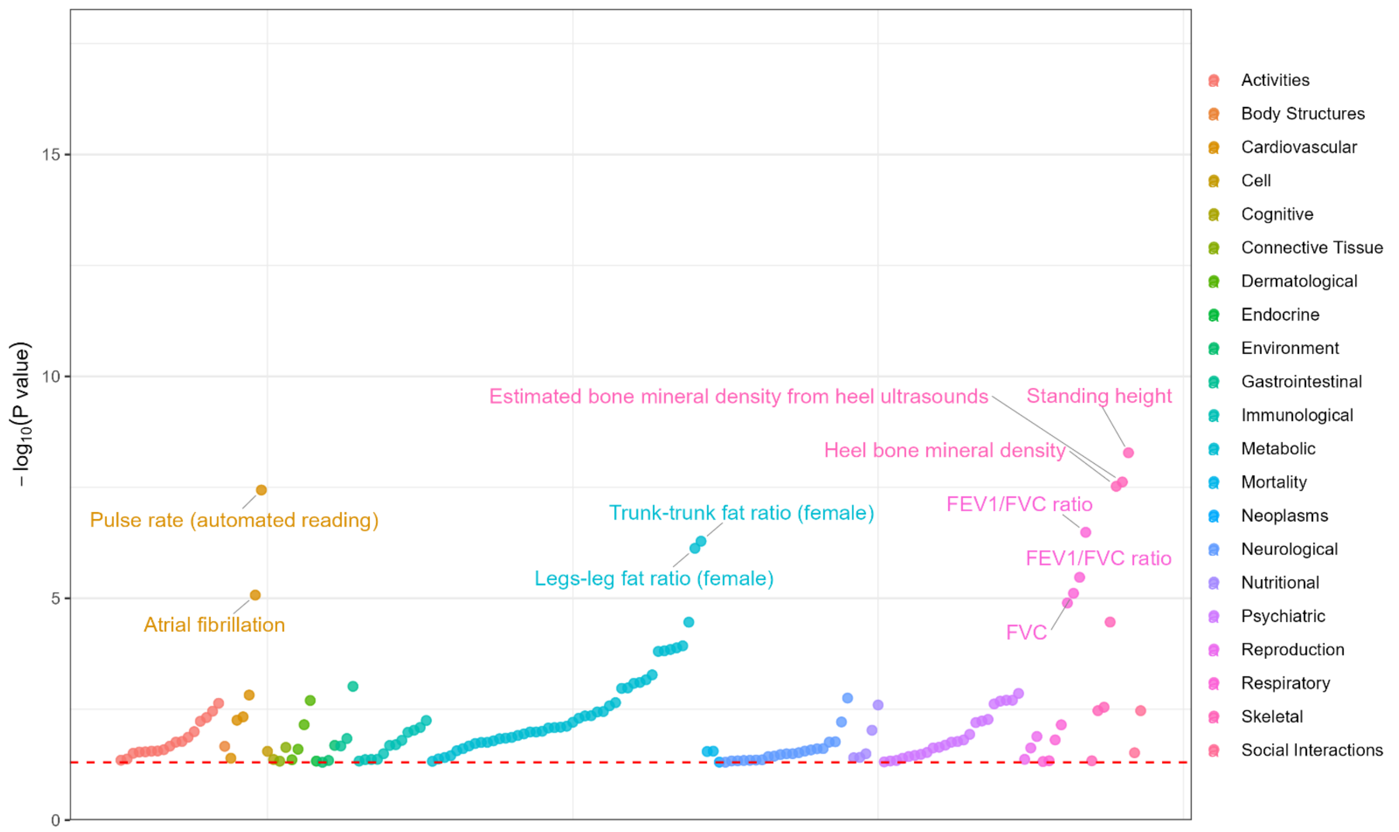

The results of the genome-wide association studies with phenotypic traits for the *NR3C1 rs41423247* variant

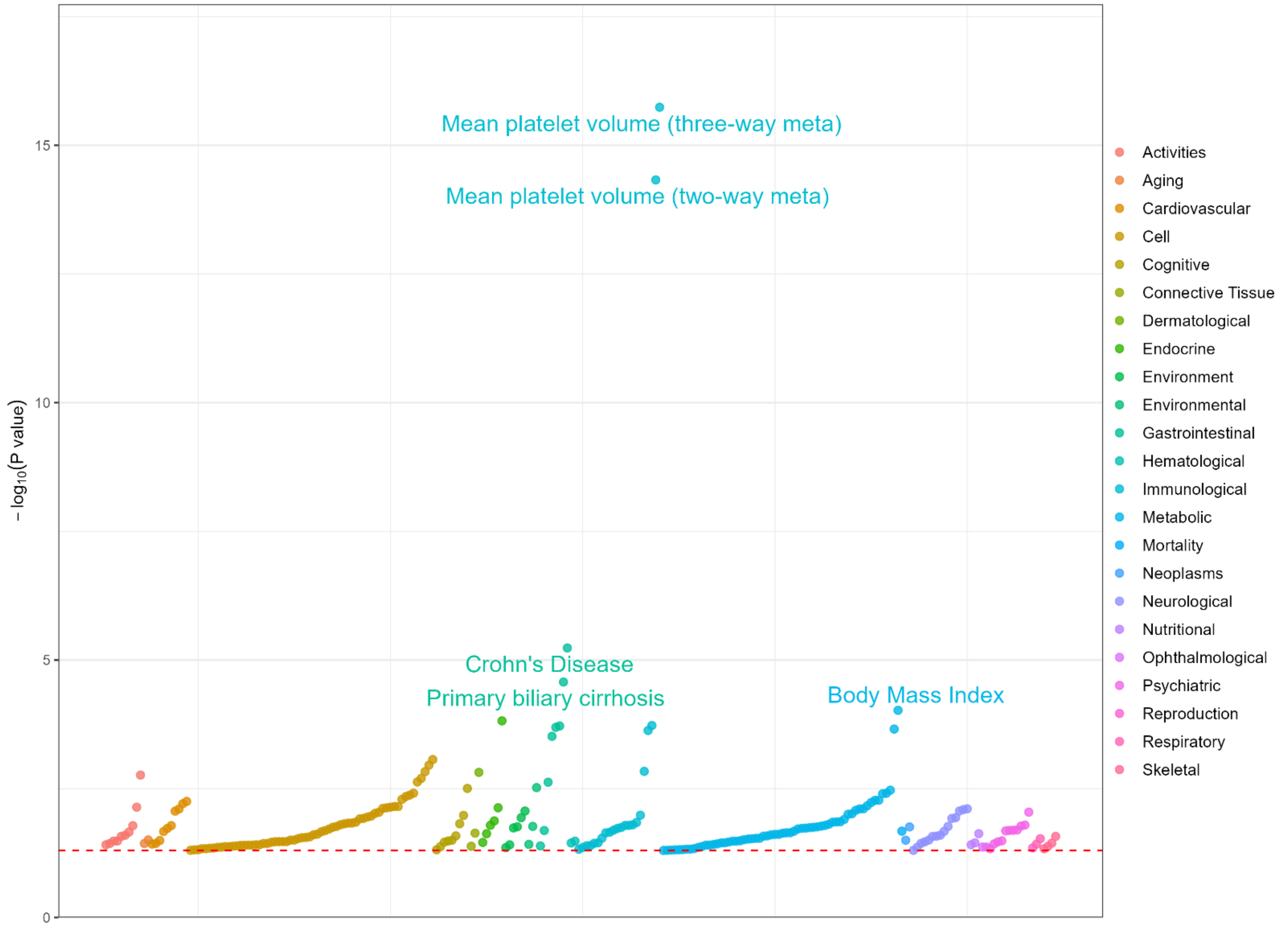

The results of the genome-wide association studies with phenotypic traits for the *IL12B rs3212217* variant

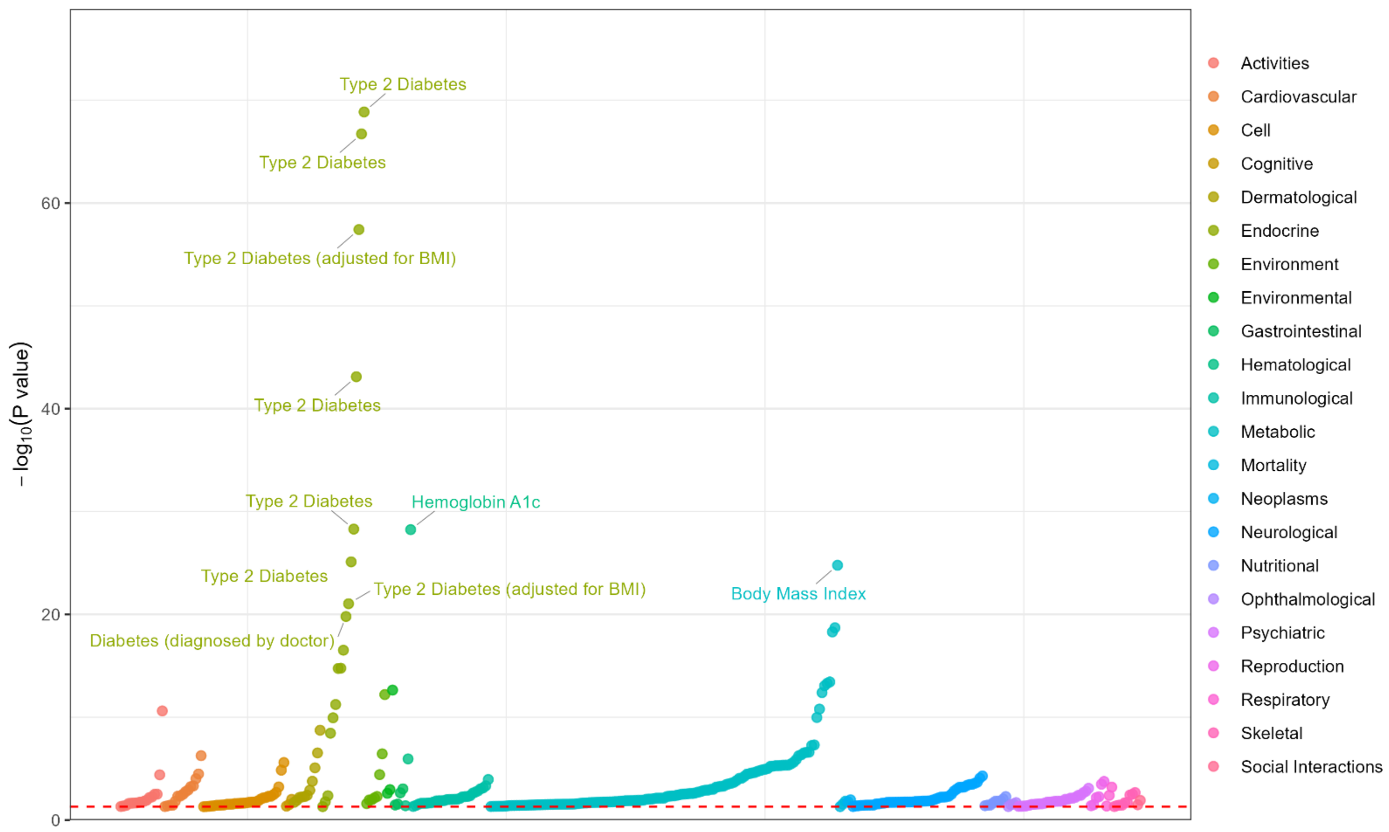

The results of the genome-wide association studies with phenotypic traits for the *CDKAL1 rs9295474* variant

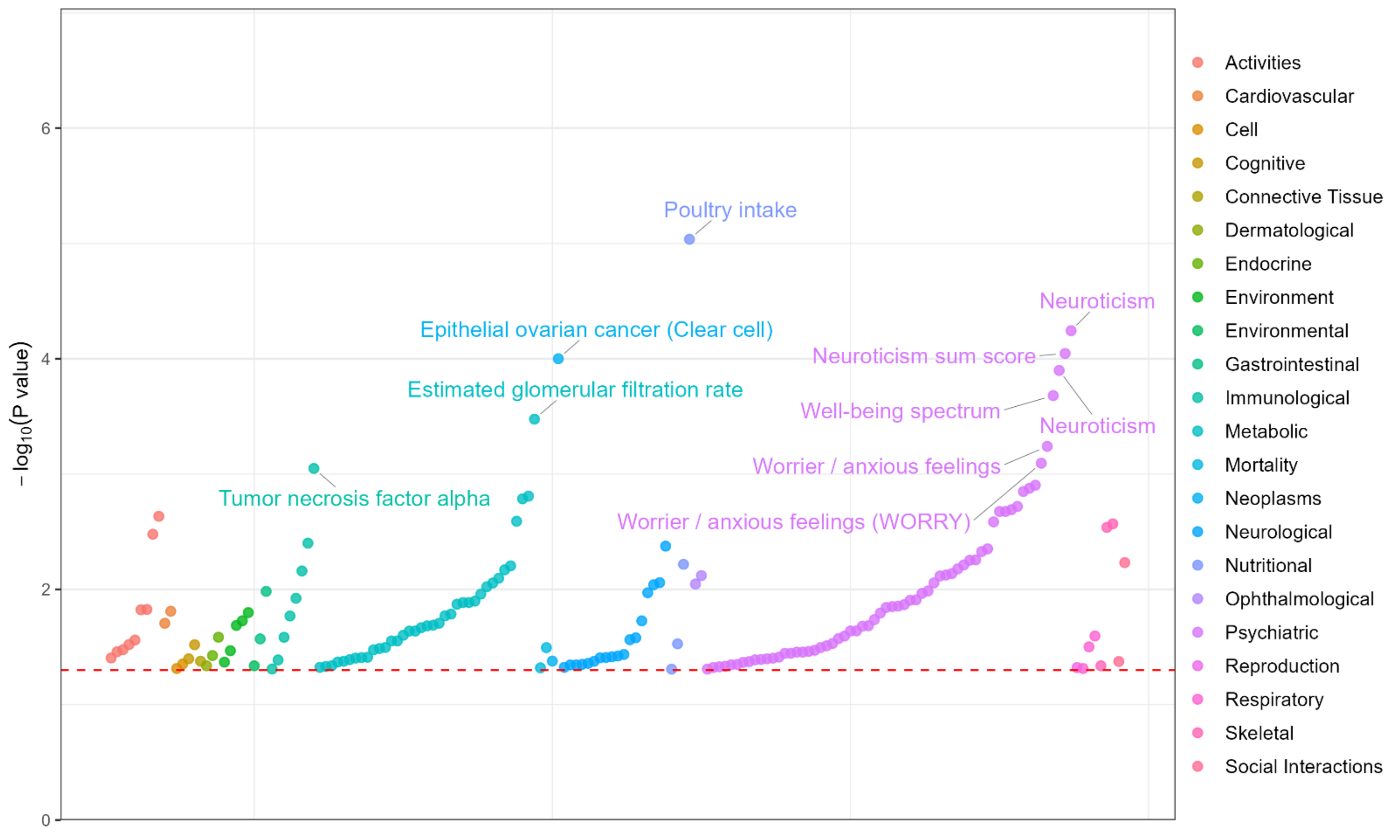

The results of the genome-wide association studies with phenotypic traits for the *HTR1B rs6296* variant

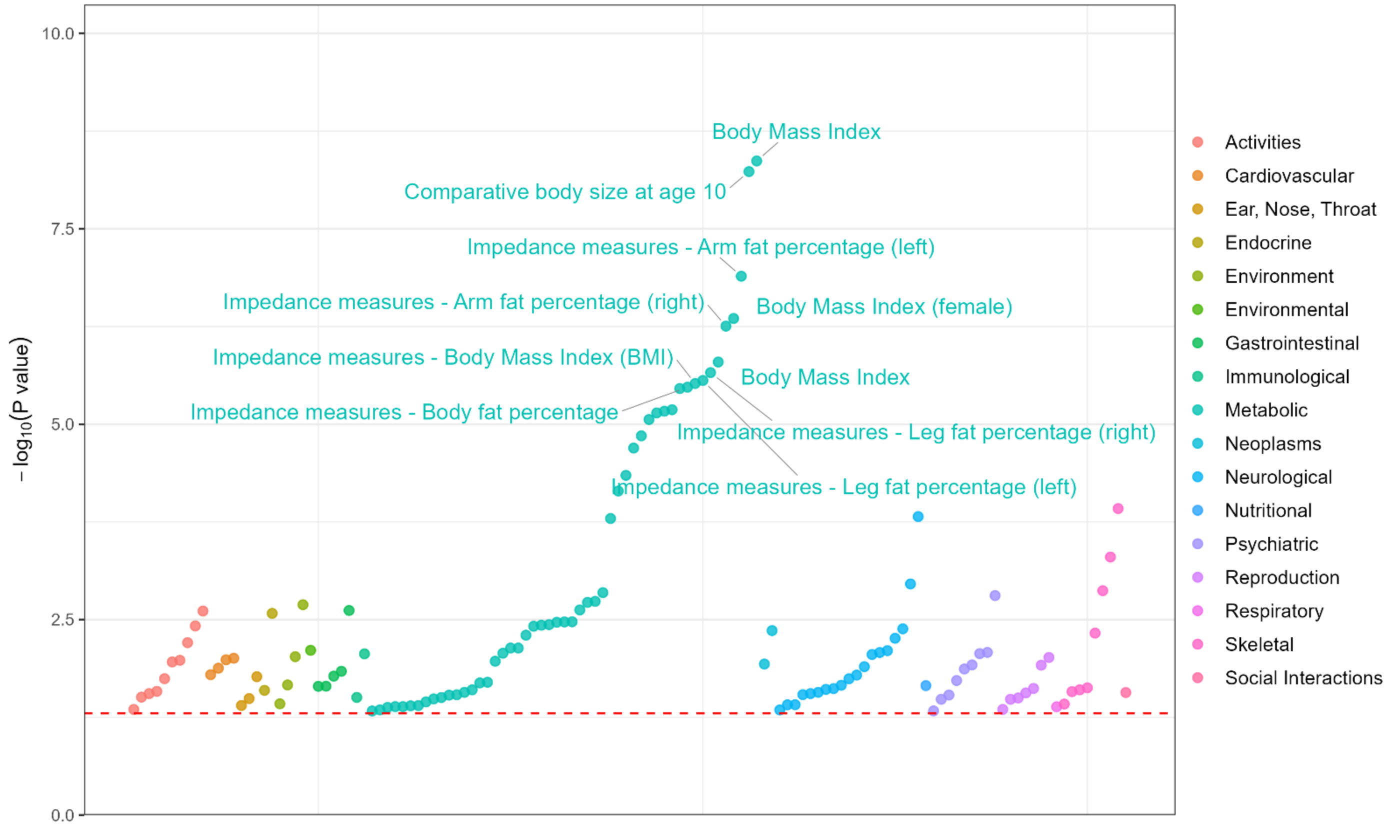

The results of the genome-wide association studies with phenotypic traits for the *NPY rs16147* variant

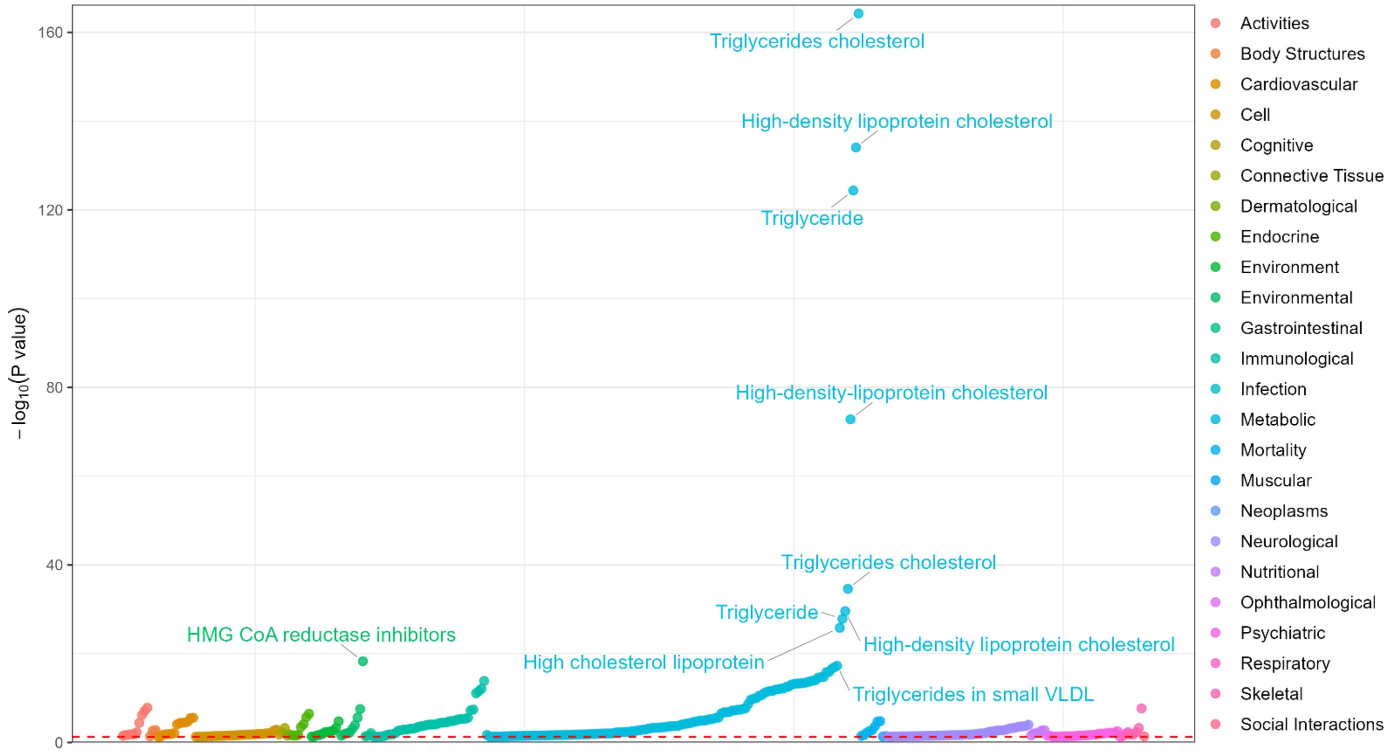

The results of the genome-wide association studies with phenotypic traits for the *LPL rs295* variant

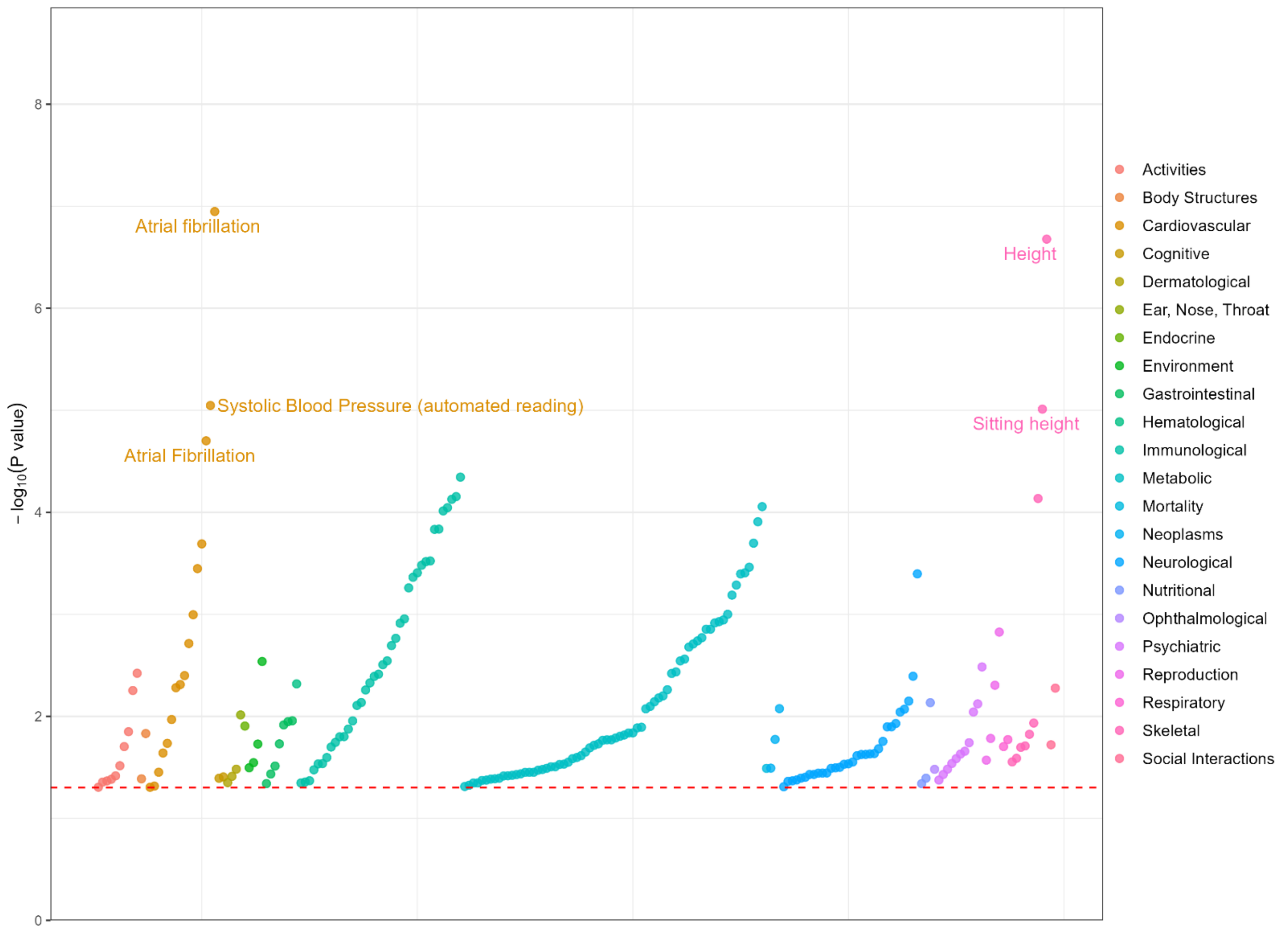

The results of the genome-wide association studies with phenotypic traits for the *SIRT1 rs3818292* variant

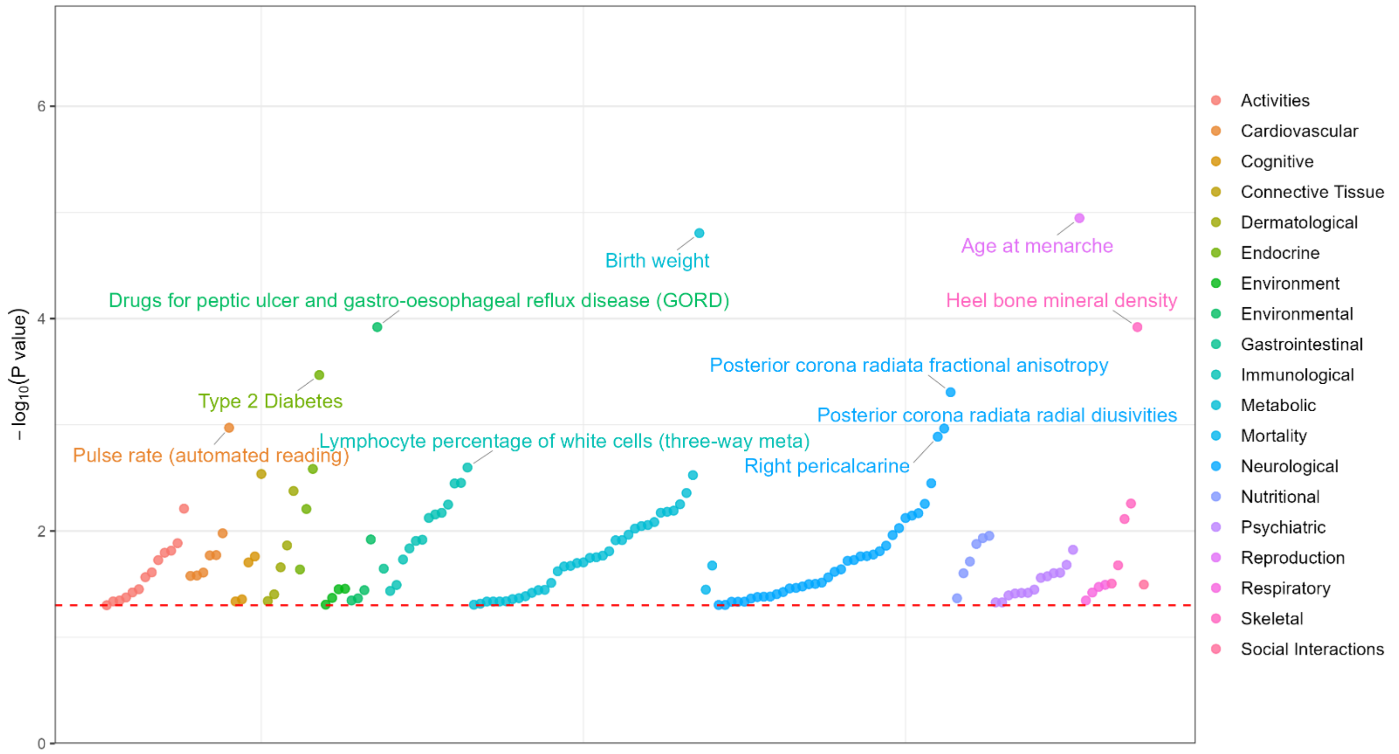

The results of the genome-wide association studies with phenotypic traits for the *ADRA2A rs1800544* variant

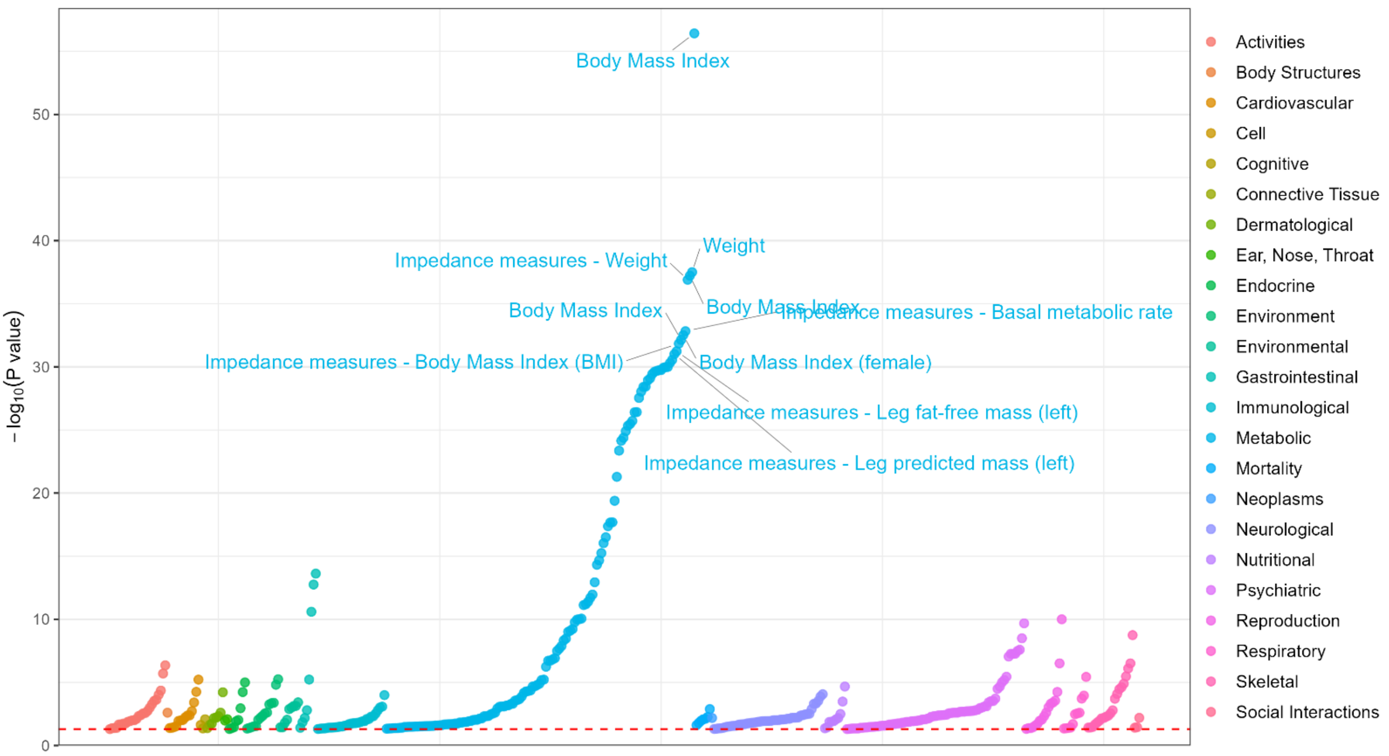

The results of the genome-wide association studies with phenotypic traits for the *BDNF rs11030107* variant

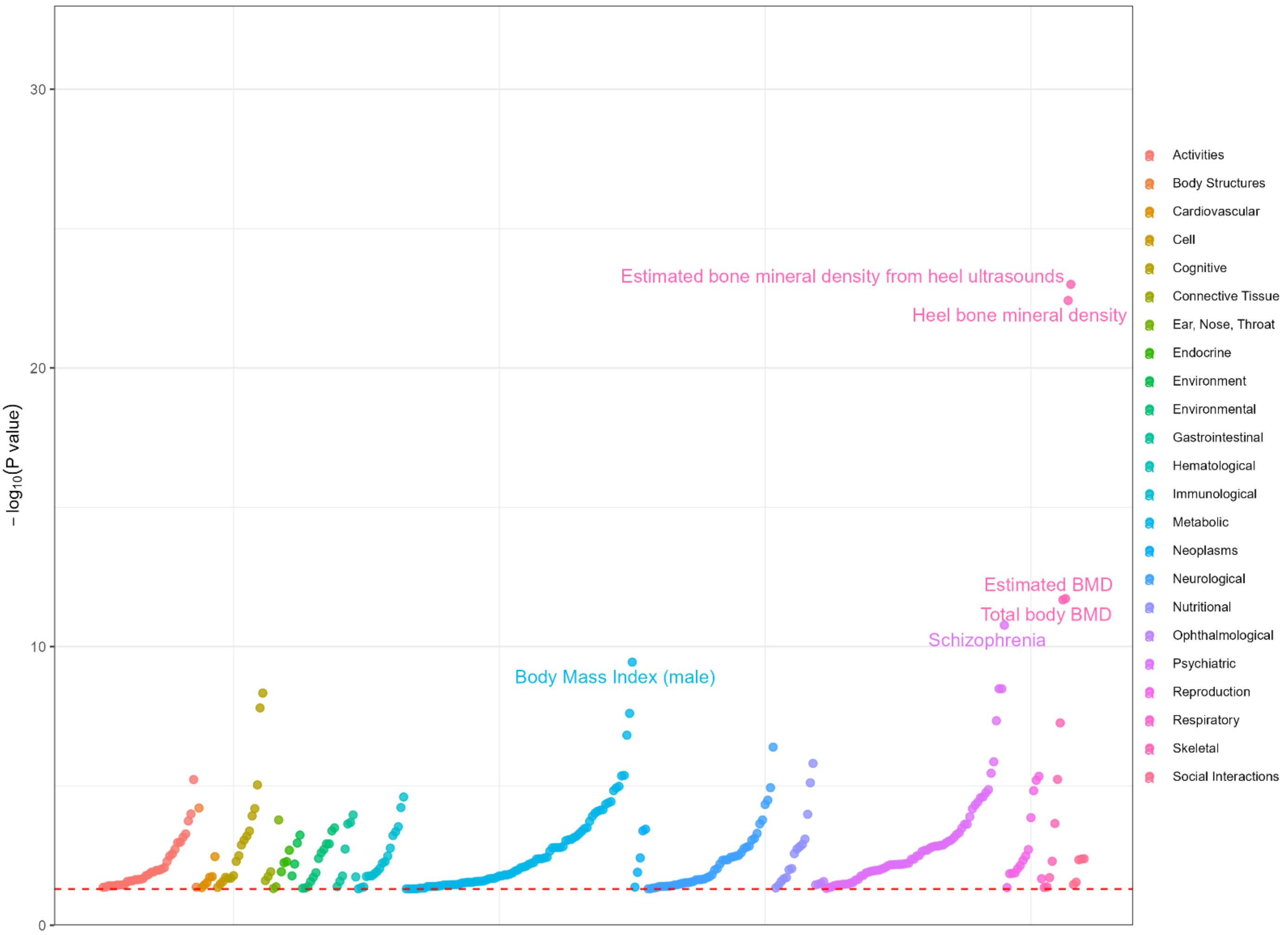

The results of the genome-wide association studies with phenotypic traits for the *CHRM4 rs2067482* variant

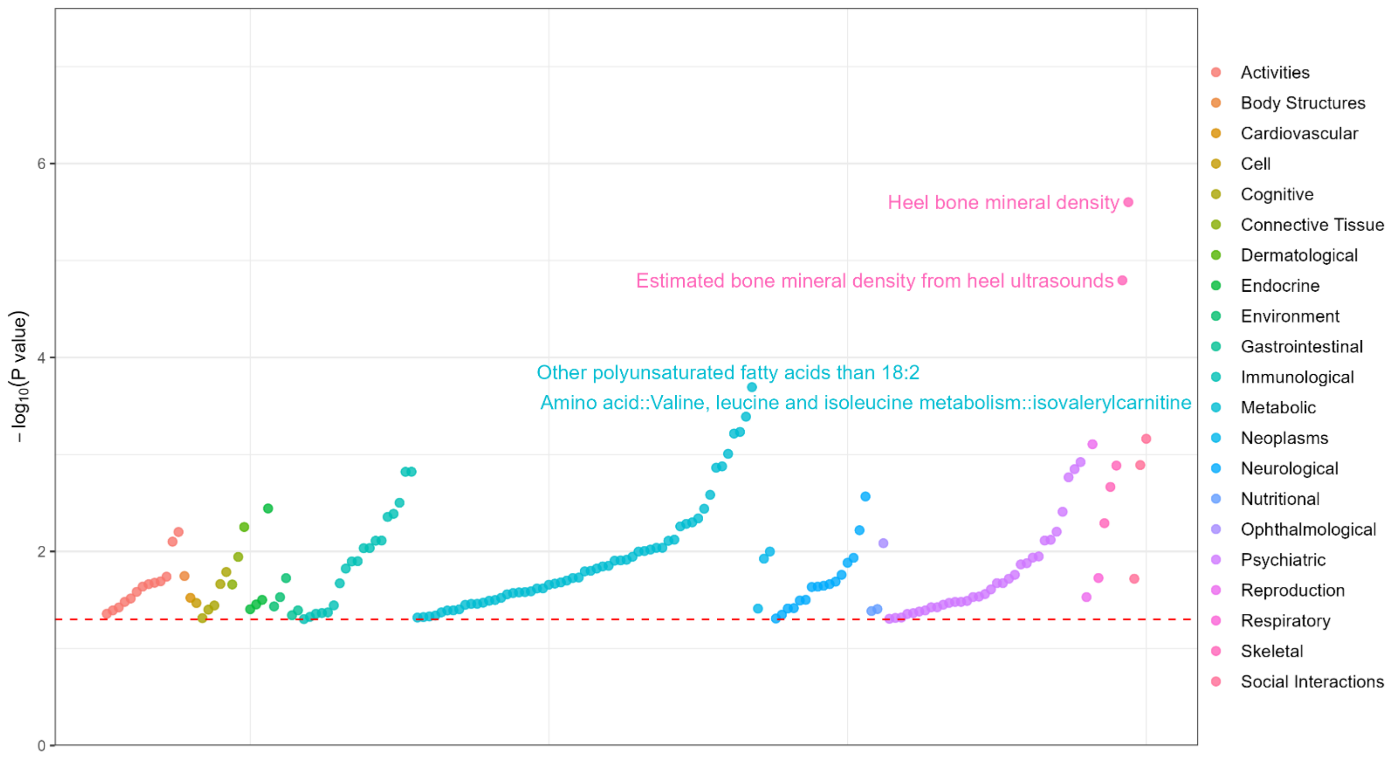

The results of the genome-wide association studies with phenotypic traits for the *CHRM1 rs2067477* variant

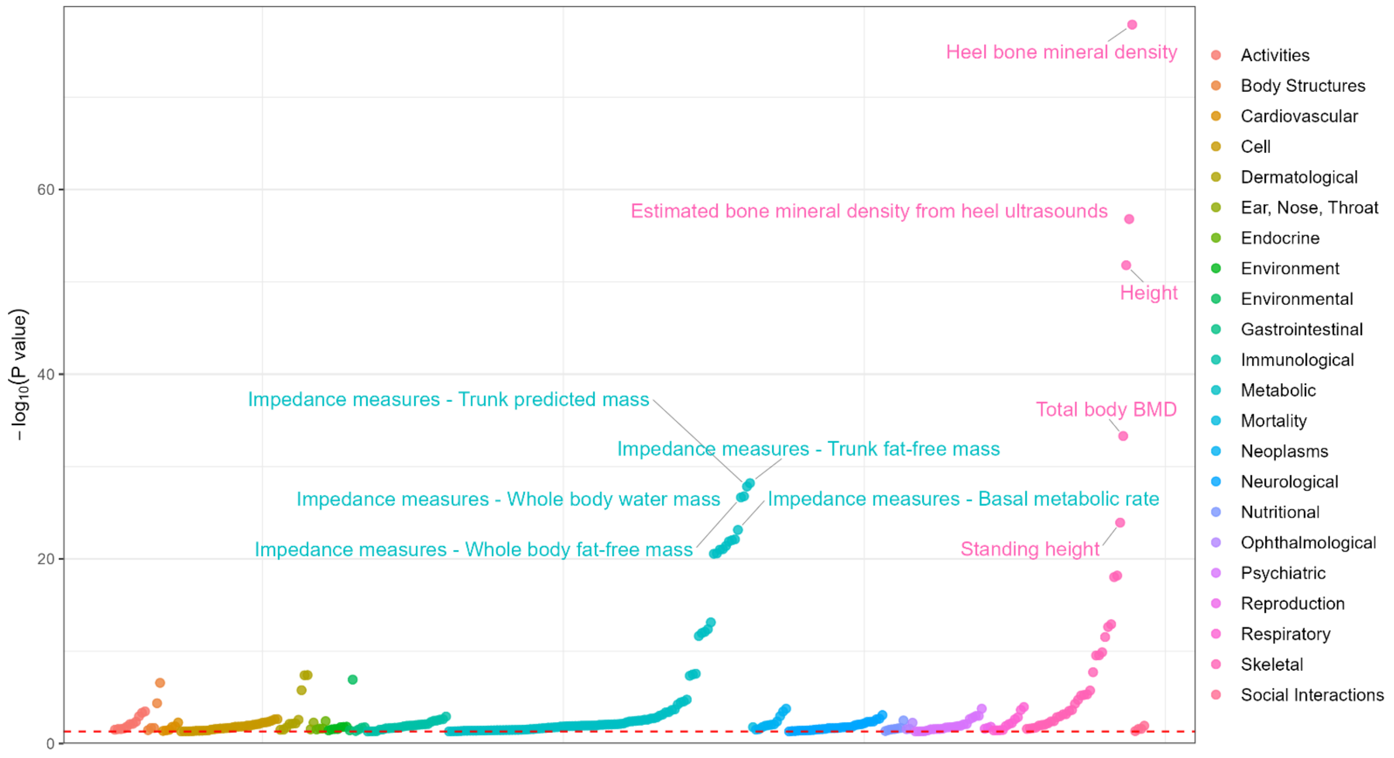

The results of the genome-wide association studies with phenotypic traits for the *LRP5 rs3736228* variant

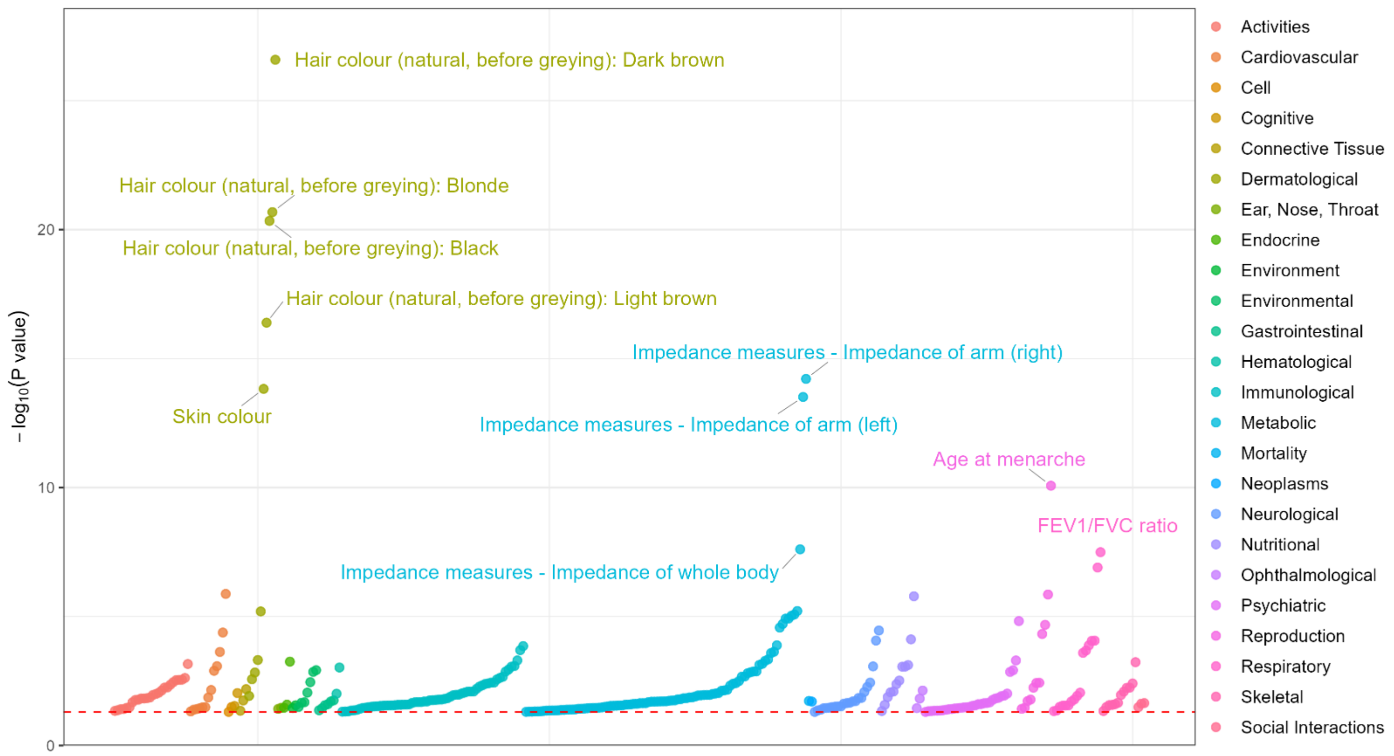

The results of the published phenome-wide association studies for the *GAB2 rs10793294* variant

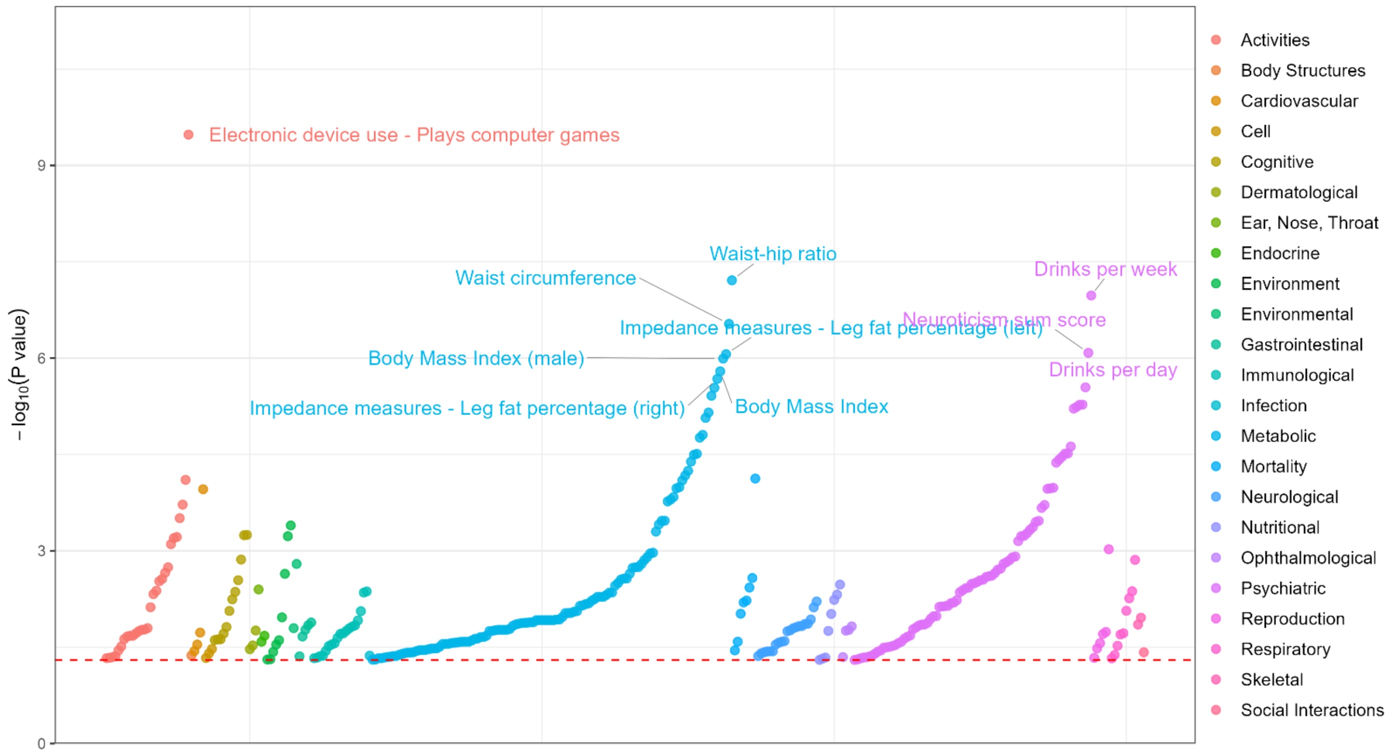

The results of the published phenome-wide association studies for the *ANKK1 rs1800497* variant

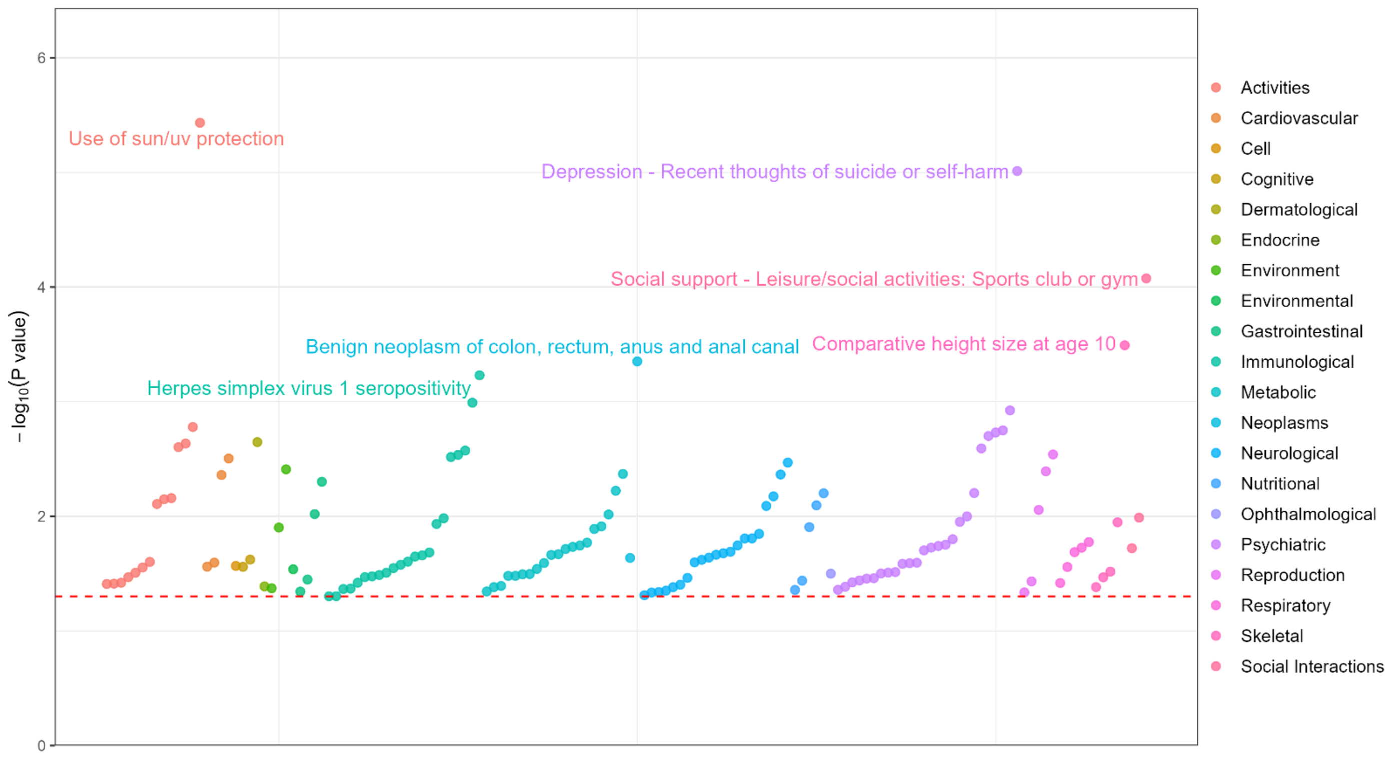

The results of the published phenome-wide association studies for the *HTR3A rs1062613* variant

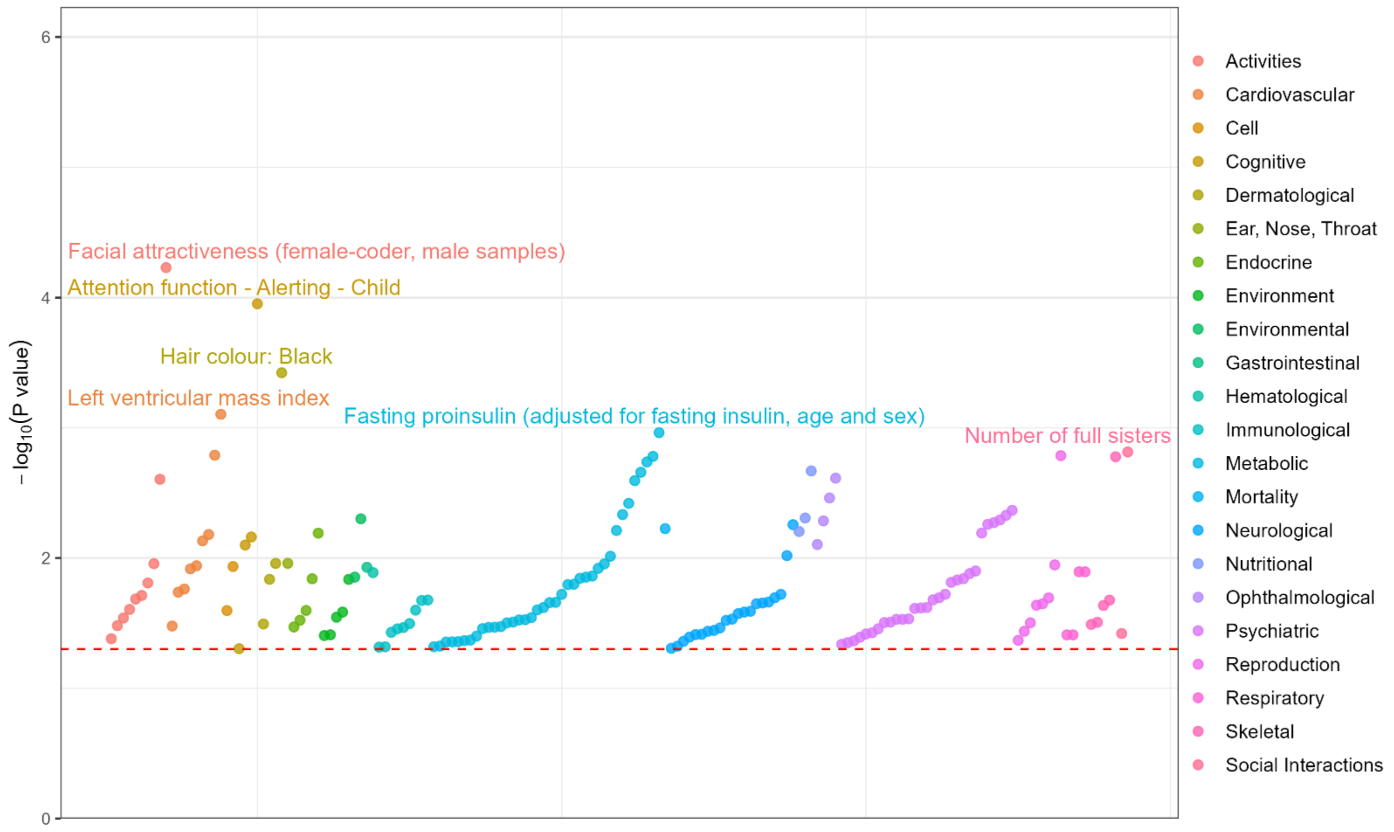

The results of the published phenome-wide association studies for the *HTR2A rs6313* variant

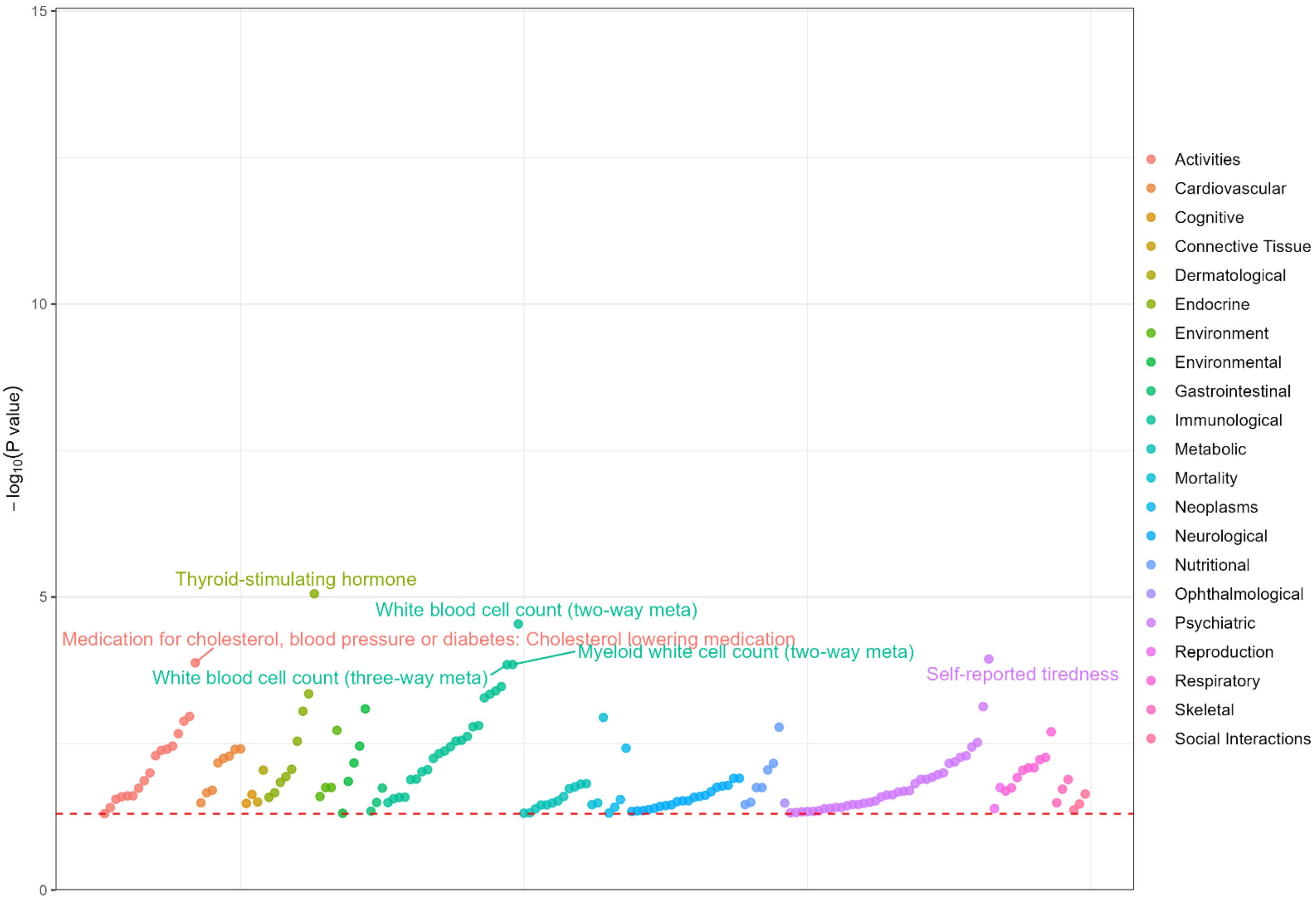

The results of the published phenome-wide association studies for the *AKT1 rs2494732* variant

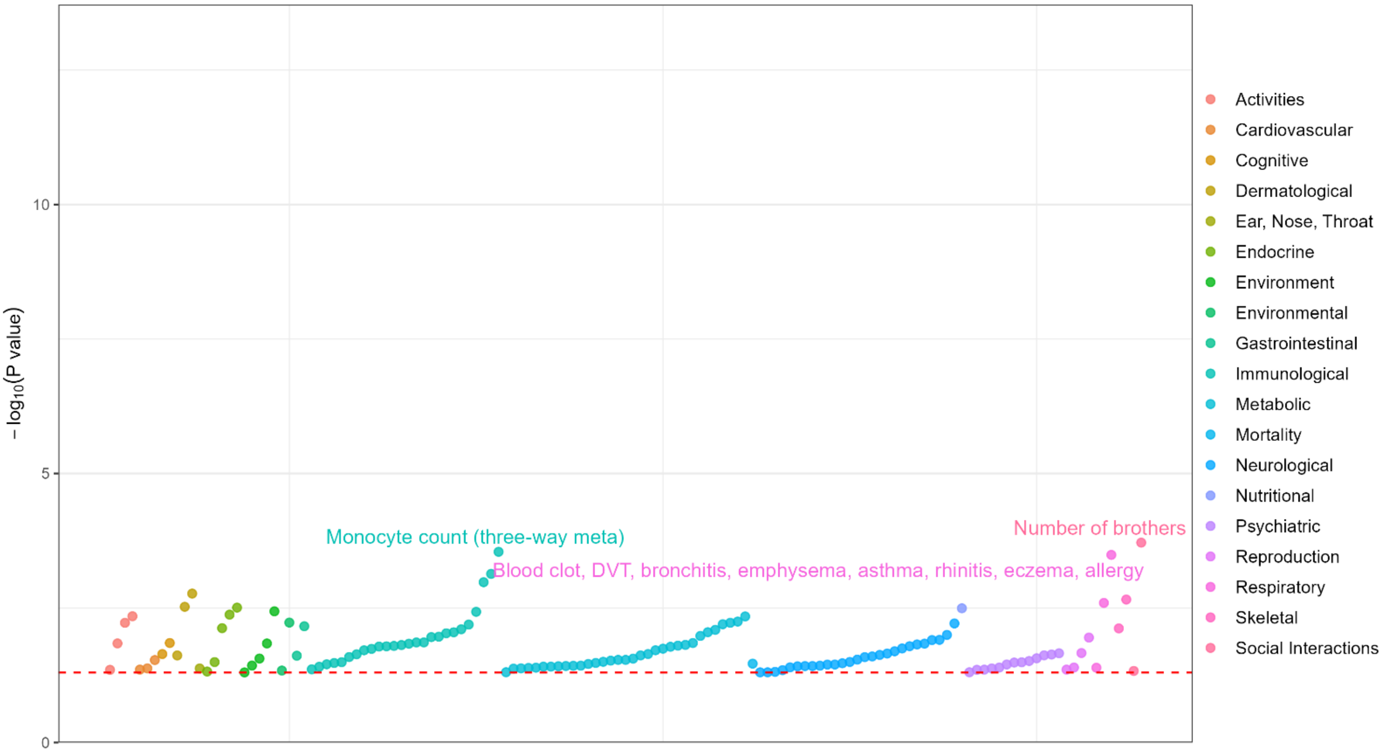

The results of the published phenome-wide association studies for the *ZBTB42 rs3803300* variant

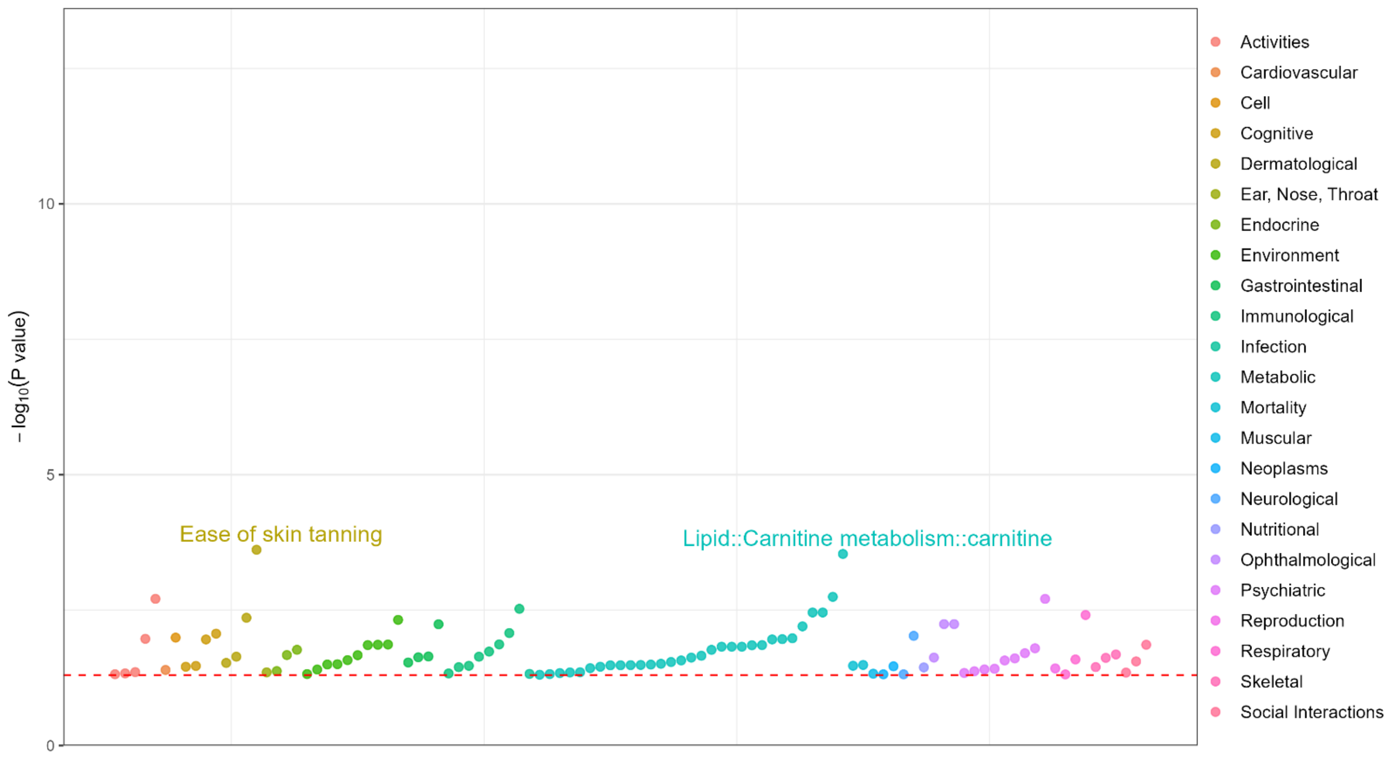

The results of the published phenome-wide association studies for the *CHRNA7 rs3826029* variant

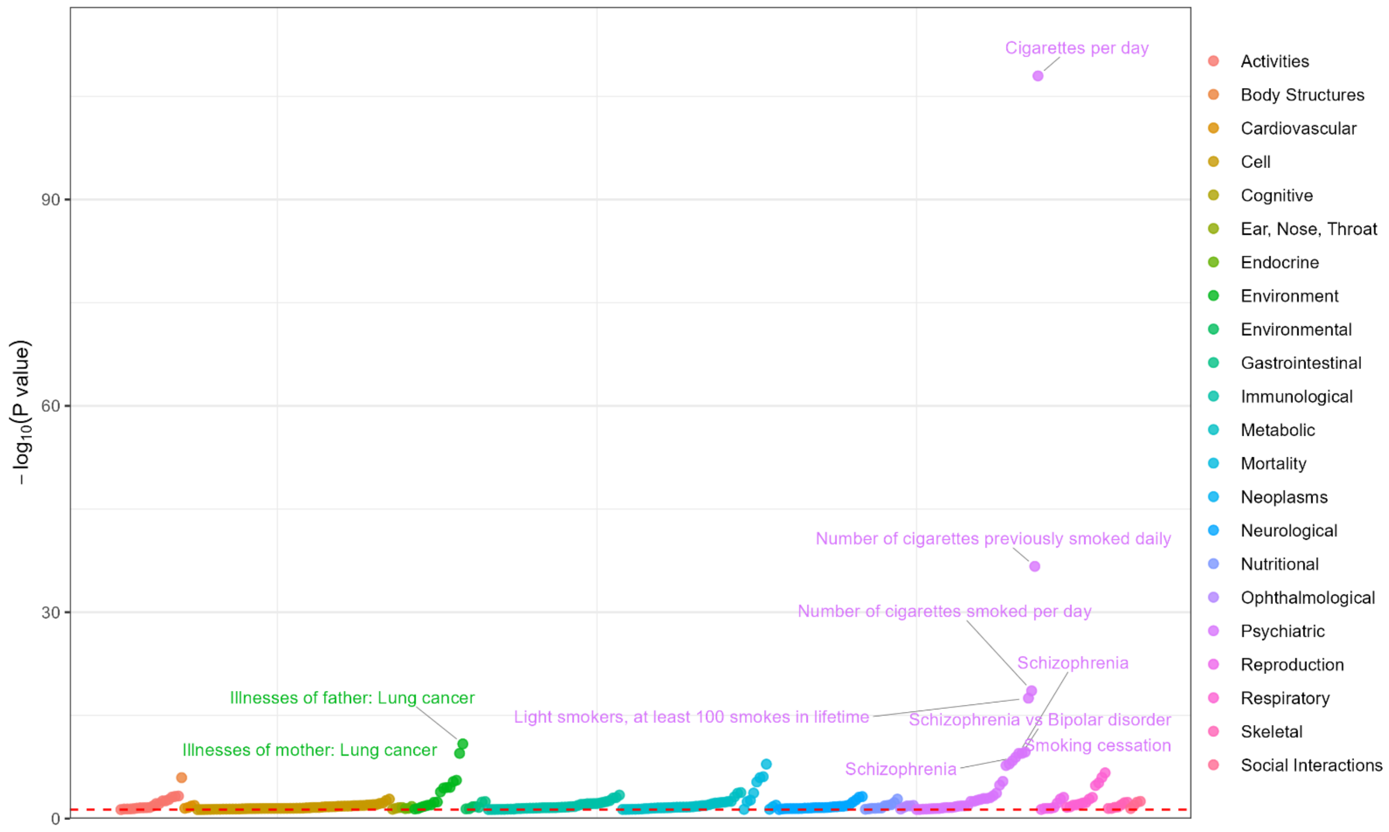

The results of the published phenome-wide association studies for the *CHRNA3 rs578776* variant

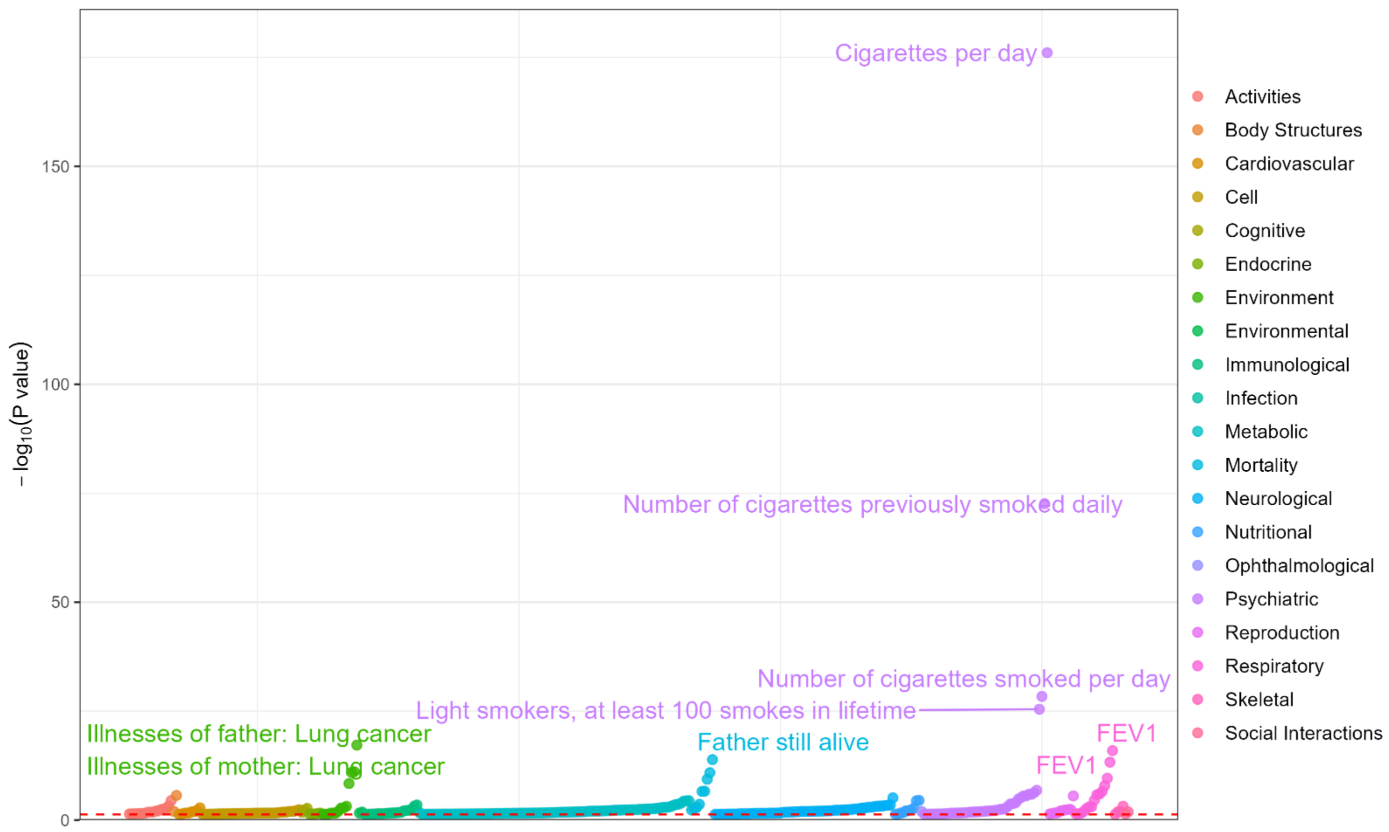

The results of the published phenome-wide association studies for the *CHRNB4 rs17487223* variant

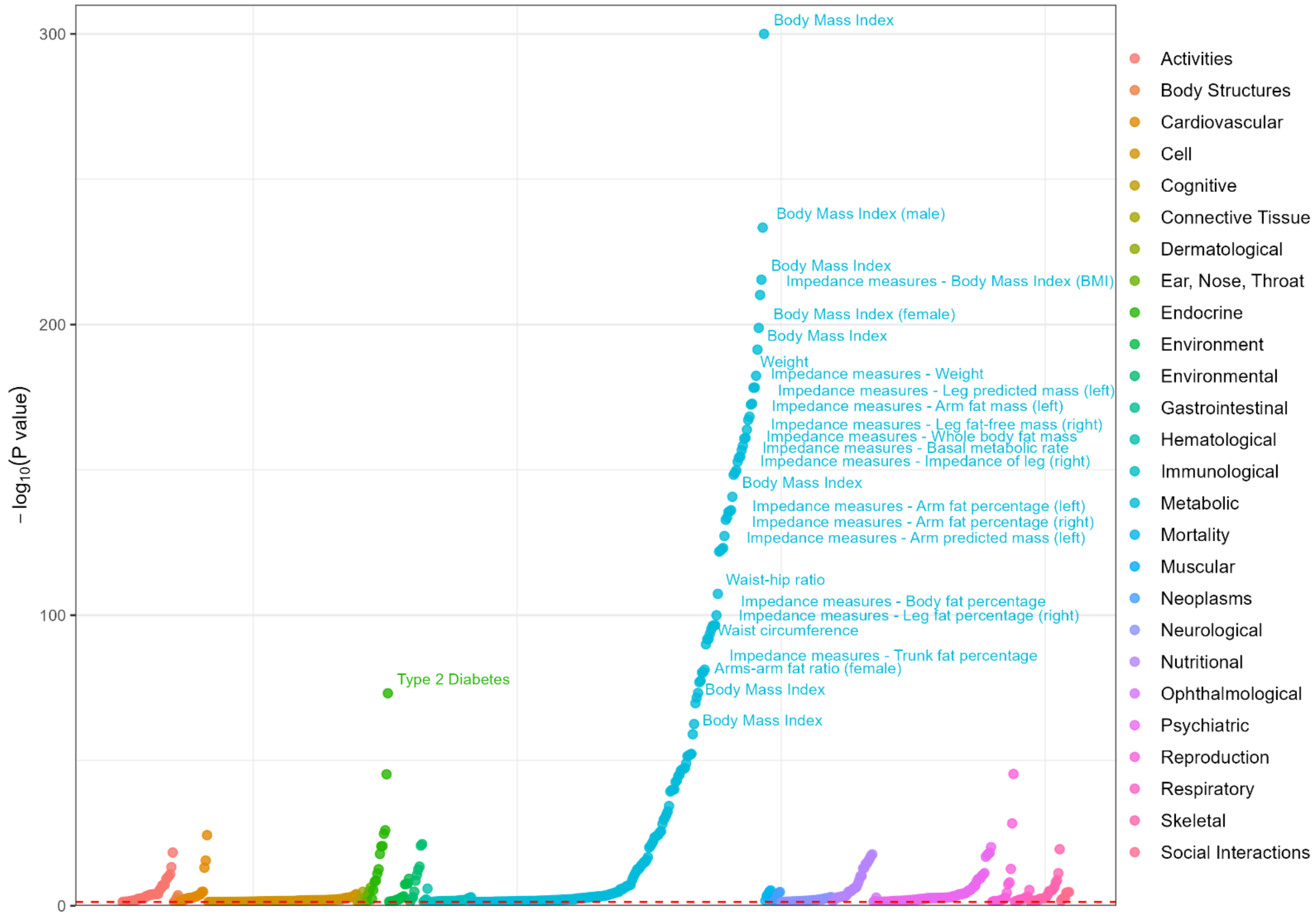

The results of the published phenome-wide association studies for the *FTO rs9939609* variant

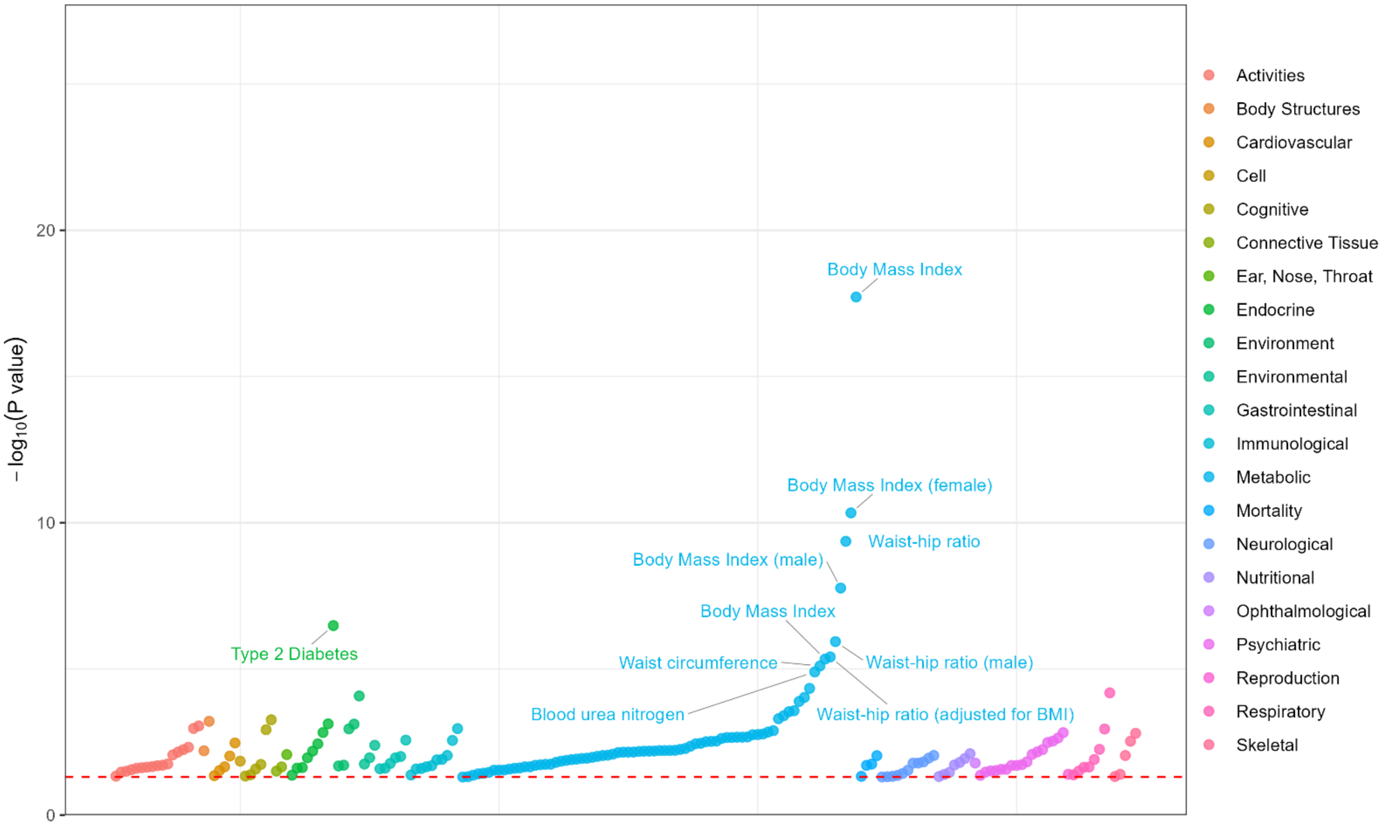

The results of the published phenome-wide association studies for the *GIPR rs2302382* variant
